# Resistance and resilience of small-scale recirculating aquaculture systems (RAS) with or without algae to pH perturbation

**DOI:** 10.1371/journal.pone.0195862

**Published:** 2018-04-16

**Authors:** Norulhuda Mohamed Ramli, Christos Giatsis, Fatimah Md Yusoff, Johan Verreth, Marc Verdegem

**Affiliations:** 1 Aquaculture and Fisheries Group, Wageningen University, Wageningen, The Netherlands; 2 Department of Biological and Agricultural Engineering, Faculty of Engineering, Universiti Putra Malaysia, Serdang, Selangor, Malaysia; 3 Laboratory of Marine Biotechnology, Institute of Bioscience, Universiti Putra Malaysia, Serdang, Selangor, Malaysia; 4 Department of Aquaculture, Faculty of Agriculture, Universiti Putra Malaysia, Serdang, Selangor, Malaysia; VIT University, INDIA

## Abstract

The experimental set-up of this study mimicked recirculating aquaculture systems (RAS) where water quality parameters such as dissolved oxygen, pH, temperature, and turbidity were controlled and wastes produced by fish and feeding were converted to inorganic forms. A key process in the RAS was the conversion of ammonia to nitrite and nitrite to nitrate through nitrification. It was hypothesized that algae inclusion in RAS would improve the ammonia removal from the water; thereby improving RAS water quality and stability. To test this hypothesis, the stability of the microbiota community composition in a freshwater RAS with (RAS+A) or without algae (RAS-A) was challenged by introducing an acute pH drop (from pH 7 to 4 during three hours) to the system. *Stigeoclonium nanum*, a periphytic freshwater microalga was used in this study. No significant effect of the algae presence was found on the resistance to the acute pH drop on ammonia conversion to nitrite and nitrite conversion to nitrate. Also the resilience of the ammonia conversion to the pH drop disruption was not affected by the addition of algae. This could be due to the low biomass of algae achieved in the RAS. However, with regard to the conversion step of nitrite to nitrate, RAS+A was significantly more resilient than RAS-A. In terms of overall bacterial communities, the composition and predictive function of the bacterial communities was significantly different between RAS+A and RAS-A.

## Introduction

Stability of a system can be described as the ability to maintain its functions under changing conditions [1,2]. In the context of recirculating aquaculture systems (RAS), water quality is an important function which relates to stability. Two properties of stability are system resistance (the ability to withstand a disturbance) and resilience (the speed of recovery of a system to its pre-disturbance state) [3–5]. In RAS, disturbances such as pH, oxygen and temperature changes may occur which will consequently affect stability.

Attramadal et al. [6] suggested that a stable RAS is linked to its stable bacterial community since bacterial communities plays a central role in maintaining water quality [7–9]. On top of that, it is known that bacteria interact with other microorganisms in the water [10,11] which may affect the stability of the bacterial community. Therefore, in this study, it is hypothesized that microalgae could improve the stability of RAS. The hypothesis was based on the shared dependency on ammonium by microalgae and nitrifying bacteria. Besides, many studies showed that the association of microalgae with bacteria could lead to a more stable system as is demonstrated by the microalgae-bacterial community in waste water treatment [12–14]. For example, Ryu et al. [13] showed that a microalgae-bacterial community was more stable and efficient in removing ammonium than nitrifying bacteria alone during thiocyanate degradation. Meanwhile, in waste treatment ponds, the existence of the microalgae population is very important for the stability of the symbiotic relationship with aerobic bacteria [12].

Therefore, the objective of this study was to assess the role of microalgae on the stability of RAS. In this study, we stressed RAS with (RAS+A) and without algae (RAS-A) by lowering the water pH from 7 to 4 for three hours. Resistance and resilience of the RAS towards the pH perturbation was calculated by measuring water quality. Additionally, the bacterial communities of RAS+A and RAS-A were compared to determine mechanisms that could explain the RAS stability. In this article, for simplification, microalgae are mentioned as algae.

## Materials and methods

### Ethics statement

The animal experiment was approved by Institute of Bioscience, Universiti Putra Malaysia research ethics and IACUC committee under the following reference number, UPM/IBS/700-3/1/IFS/6384000(R22.1).

### Recirculating aquaculture system

The experiment was conducted at the Laboratory of Marine Biotechnology, Institute of Bioscience, Universiti Putra Malaysia. In the experiment, eight recirculating aquaculture systems (RAS) were used. The RAS had been in operation for 10 weeks before this experiment was conducted.

**Fig 1:**
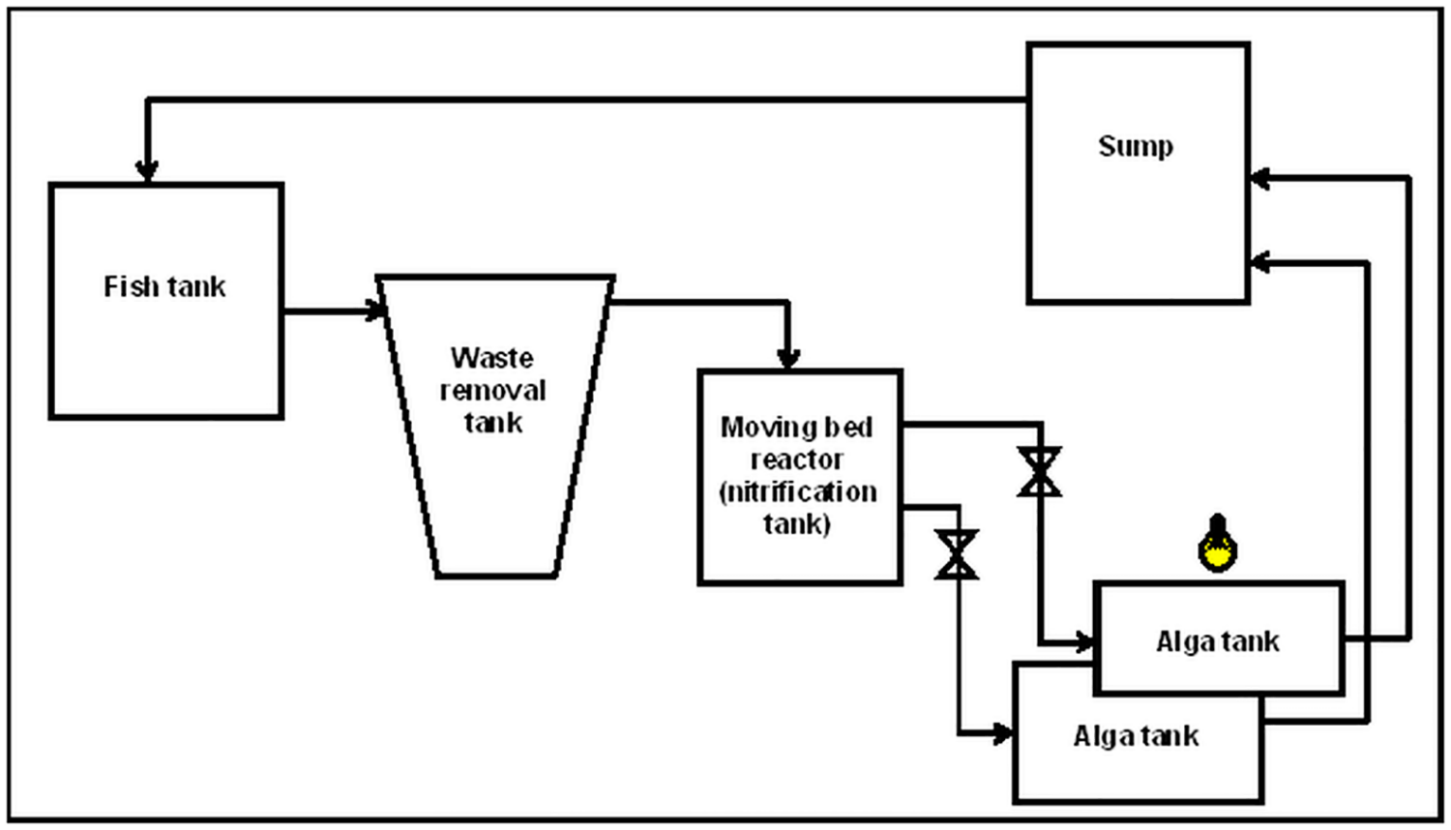
Conceptual experimental set-up of recirculating aquaculture system (RAS) with two algae tanks. The total system volume for the RAS was 260 L.

The four RAS with algae (RAS+A) consisted of a fish tank (65 L), a hydro-cyclone for fecal solids removal, diameter 30 cm (effective volume: 42 L), a moving bed reactor (30cmX 30 cmX 30 cm) (effective volume: 14 L) with bio-filter media (Ai.M K1 Biological Filter Media, size 1 cm, Malaysia Fish Harvest), two tanks units with algae (30cmX 30 cmX 30 cm) (14 L each) and a sump (112 L) (Fig 1). The moving bed reactor was conditioned and had been in operation for ten weeks before the experiment started. The four RAS without algae (RAS-A) had the same configuration as RAS+A except that the tank for algae was filled with water only. The flow rate from the fish tank to the sedimentation tank and the moving bed reactor was 6 L min^-1^. Water from the moving bed reactor flowed into two algae tanks, each receiving half of the water flow (3 L min^-1^). Water from the algae tanks flowed to the sump from where it was pumped to the fish tank.

A periphytic algae *Stigeoclonium nanum* was incorporated in the RAS. This periphytic algae was chosen instead of planktonic algae so that the density of suspended algae in the RAS could be kept sufficiently low to avoid clogging pipes and bio-filters. The algae was isolated from the university’s aquaculture experimental facility. Our previous study indicated that *S*. *nanum* preferred ammonium than nitrate; therefore, its inclusion in RAS would improve total ammonia nitrogen (TAN) removal [15]. The algae tanks were maintained in 24 hours light of 55–60 μmol photons m^-2^ sec^-1^ and were aerated. RAS water temperature was maintained at 26–28 °C, pH at 6.8–7.0, dissolved oxygen at 7.0–8.0 mg L^-1^, and conductivity at 2500–3000 μS cm^-1^ (slightly below 1.5 ppt salinity), during the experiment.

### Experimental design

The experiment consisted of a period before and after stress. Before the stress (d-1), eight RAS systems were divided over two treatments, RAS with (RAS+A) and RAS without algae (RAS-A). The next day (day 0), two replicates from each treatment were subjected to a stressor (+S) and the other two replicates became the control (no stressor, -S). The stressor that was applied was gradually lowering the pH from 7 to 4 within a period of three to four hours, followed by 3h at pH 4, and thereafter restoring the pH back to 7 within a period of two to three hours. Hence, the whole operation of applying the pH stressor lasted eight—ten hours in total.

### Experimental procedure

During the experiment, each RAS had 2200 g red Nile tilapia (*Oreochromis niloticus*). The fish were bought from a commercial fish farm at Puchong, Selangor, Malaysia (Atlantys Hatcheries Sdn. Bhd.). The fish were fed twice a day with a 40% protein diet at 1.8% body weight per day (crude protein 43%; fat 6%; and moisture 12%—Starfeed 9971, Star Feedmills Sdn. Bhd., Malaysia).

Before the stressor was applied to the RAS, the fish were removed from the system and restocked after the pH was raised back to pH 7. During handling, they were anasthesized using 0.4 gL^-1^ of tricaine methanesulfonate (TMS, Crescent Research Chemicals, Phoenix, Arizona, USA) buffered with 0.8 gL^-1^ of sodium bicarbonate.

The pH was lowered from 7 to 4 (S1 Fig) by gradually adding 3 ml hydrochloric acid (12 N) at a time. After 3 hours at pH 4, the pH was restored back to 7 gradually by adding 1.0 g sodium bicarbonate (NaHCO_3_) at a time. Hydrochloric acid and NaHCO_3_ addition were done in the sump. The next morning after the stressor had been applied, TAN increased in some RAS. Therefore, partial water exchange (8–16% from total water volume) was applied to neutralize the effects from lowering the pH and to keep the TAN level <3 mgL^-1^ (S2 Fig). Water was discharged from the bottom of waste removal tank (hydro-cyclone). During discharge, the hydro-cyclone was disconnected in such a way the other system component maintained functioning. Tap water which was dechlorinated and stored in a reservoir was used to refill the RAS after water discharge. The same water discharge procedure was practiced in all treatments.

During the experiment, temperature, pH, dissolved oxygen, and electrical conductivity levels were monitored daily using a water quality probe (Aquaread, 2000, United Kingdom). TAN level was monitored in the system one day before stress (d-1) until day 20 after stress (d20), with the pH stressor being applied on day 0. Nitrite-N (NO_2_-N) and nitrate-N (NO_3_-N) were monitored on days -1, 6, 13, and 20. Analysis of TAN was done using the phenate method [16]. Except for day 8 until 11, TAN was measured using API ammonia test kit (Mars Fishcare North America, Inc., USA) due to technical problem with spectrophotometer. NO_2_-N and NO_3_-N concentrations (mgL^-1^) were analyzed using ion chromatography. Chromatographic analyses were performed using a Metrohm model 882 Compact IC Plus (Metrohm, Herisau, Switzerland) with suppressor module at room temperature. Bacterial community analysis was performed on d-1 and d20. For water quality and bacterial community analysis, one litre water was sampled in the fish tank (Lf), the algae tank (La) and the biofilter (Lb).

The volume of the algal tank was 14 L with 15.6 cm depth. Most of the algae attached on the reactor walls and some were floating in the tank. During the experiment, the chlorophyll-a content of the algae was maintained at 5.8 ± 0.6 mg per RAS (22.3 μg L^-1^) by maintaining the same area covered by the algae by scrapping the old cells that were attached on the tank walls weekly. The outlet of the algal tank was equipped with a strainer to prevent the algae from flowing to the sump.Measurement of chlorophyll-a was done weekly by sampling the area covered by the algae (APHA, 1999).

### Microbial analysis (DNA extraction, PCR, and 16 S rRNA metagenomic)

The bacterial composition in the fish, biofilter (moving bed reactor) and algae tanks was determined. The sample was filtered using membrane water filters (isopore polycarbonate membrane filter, 0.22 μm pore size, Merck, New Jersey, USA).

DNA was isolated from the membrane water filters using Macherey Nagel genomic DNA extraction kit (Nucleospin^®^ Soil, Düren, Germany) following instruction by the manufacturer. The membrane was cut into small pieces and 250–350 mg of the membrane was used. The sample was homogenized and lysed in lysis buffer (Buffer SL2) by 15 minutes vortexing using a bead tube (Nucleospin^®^ Bead Tube). After lysis, the sample was incubated in buffer SL3 for 5 minutes at 0–4 °C and then centrifuged at 11000 x g for one minute to precipitate the contaminants. After that, supernatant was collected and inhibitors were removed using inhibitor removal column (Nucleospin^®^Inhibitor Removal Column). The filtrate which contained DNA was bound, washed, dried and eluted. DNA was quantified using NanoDrop spectrophotometer (Thermo Scientific Nanodrop, NanoDrop Technologies, Wilmington, DE, USA) and visualized using 0.8% agarose gels using a nucleic acid gel stain (GelRed^™^ Nucleic acid gel stain, Biotium, California, USA). DNA was stored at -20 °C until analysis.

For 16S rRNA metagenomic analysis for day -1, DNA from four replicates was pooled, resulting in six samples. For day 20, DNA from two replicates was pooled, resulting in 12 samples. 16S rRNA metagenomic analysis was done using Illumina MiSeq according to the protocol described by the manufacturer (Illumina Inc, San Diago, USA). Briefly, the workflow included 16S library preparations, library quantification, normalization and pooling, library denaturing and sample loading, and finally, sequencing and data analyzing.

For the library preparation, two-staged PCR was involved. First, target fragments of Microbial 16S ribosomal RNA gene were amplified from V3 and V4 regions from the extracted DNA by PCR using primers suggested in the protocol [17]. PCR cycle condition was 95 °C for 3 min, followed by 25 cycles of 95 °C for 30 s, 55 °C for 30 s and 72 °C for 30 s and then a final extension at 72 °C for 5 min. Then, samples were cooled to 4 °C. After that, PCR clean-up was run to purify the 16S V3 and V4 amplicon from free primers and primer dimer species using AMPure XP beads. In the second PCR, dual indices and Illumina sequencing adapters were run using Nextera XT Index Kit. PCR cycle condition was 95 °C for 3 min, followed by 8 cycles of 95 °C for 30 s, 55 °C for 30 s and 72 °C for 30 s and then a final extension at 72 °C for 5 min. Then, samples were cooled to 4 °C. Finally, a second PCR clean-up was done to clean-up the library before quantification. Library validation was done using Bioanalyzer DNA 1000 chip to verify the size. After library quantification, normalization, and pooling, the library was denatured and ready to be loaded into the MiSeq system for sequencing.

Open reference operation taxonomic unit (OTU) picking work flow was used to search the reads generated from MiSeq sequencing. Pre-filtration of reads was done in order to discard the sequences which did not represent the targeted marker gene. After that, sequences were clustered using UCLUST v1.2.22 in parallel by a closed-reference OTU picking workflow against the reference database (Greengenes 13_8) at percent identity 97%. The reads that were matched to the reference sequence at greater than or equal to 97% identity were assigned to the OTU defined by the reference sequences. Next, a random subsample (0.1%) of the sequences that failed to match the reference sequence (0.1% from total sequences) was clustered *de novo*. The cluster centroids for all resulting OTUs were used to define a new reference OTUs. The sequences which were not included in the random subsample went through an additional round of closed-reference OTU picking workflow against the new reference OTUs. Finally the reference OTU and the new references OTUs were combined into a single OTU table.

Functional analysis of OTUs derived from 16S rRNA metagenomic sequencing was performed using PICRUSt (phylogenetic investigation of communities by reconstruction of unobserved states) [18]. In this analysis references which were clustered de novo were removed and only those that have Greengenes OTU identities were further analyzed.

### Data processing and statistical analysis

Before statistical analysis, water quality data were checked for normality and equal variances. For water quality, a three-way ANOVA repeated measure analysis with algae (+A and -A), location (La, Lb, and Lf) and stressor (+S and -S) was used. TAN conversion rate was calculated using the formula;

$$TAN\;conversion\;rate=({TAN\;produced}_{day=i-1}-{TAN\;measured}_{day=i})÷day.$$

TAN produced was calculated based on Ebeling [19]. TAN converted was equal to nitrite produced and used to calculate nitrite conversion rate using the same formula for calculating TAN conversion rate.

Resistance and resilience which were based on the TAN and nitrite conversion rate were calculated following Orwin and Wardle [2]. The results were compared between stressed RAS+A and RAS-A using a one-way ANOVA repeated measure analysis.

From the result of Illumina sequencing, Chao1 richness was calculated. To allow fair comparison between samples, random number of sequences for each sample was selected to count based on the minimum reads (315,930 reads) and used for calculation. For d-1, algae and location factors were compared and for d20, algae, location and stressor factors were compared. ANOVA test on main factor design was performed using Statistical software SPSS (IBM SPSS Statistics, version 20).

From Illumina sequencing, relative abundance of OTUs was square root transformed and the similarity analyses between samples were performed using Bray-Curtis similarity. Then, Principle Component Analysis (PCO) was performed to represent the samples in a low dimensional space in a way that relative distances of all points represent the relative dissimilarities of the samples as measured by the Bray Curtis index.

To examine the significant differences between treatments, permutation based multivariate ANOVA (PERMANOVA) was used. P-value which derived from Monte Carlo algorithm was used when the possible number of permutations was 60 and below. Samples from d-1 were analyzed using two factors; “algae” (two levels; +A and -A; fixed) and “location” (three levels; Lf, Lb and La; fixed). Samples from d20 were analyzed using three factors; “algae” (two levels; +A and -A; fixed), “location” (three levels; Lf, Lb and La; fixed) and “stressor” (two levels; +S and -S; fixed). Similarity percentage analysis (SIMPER) was used to show which OTUs contributed to the difference of bacterial community between algae factor. Cluster of orthologous genes (COG) which were derived from PICRUSt analysis were analyzed using the same procedure for analyzing the OTUs.

Statistical analyses (Bray-Curtis similarity, PCoA, PERMANOVA, and SIMPER) were performed using the multivariate statistical software package Primer V6 Permanova+ (Primer-E Ltd, Plymouth, UK).

## Results and discussion

### Water quality

Some of the general benefits of algae inclusion in an aquatic system are; 1) to reduce the pH fluctuations due to extraction of carbon dioxide during photosynthesis; 2) to reduce TAN, NO_2_-N and NO_3_-N concentration in the water by algae assimilation and; 3) to regulate dissolve oxygen in the water [20]. Low biomass of *S*. *nanum* observed in this experiment was probably due to the low light used in the study. However, effects of the low biomass were still observed on NO_3_-N level, on the resilience after the pH perturbation, and on the bacterial community of the RAS.

This experiment was a part of a larger experiment which studied the effect of algae inclusion under normal condition and under stressed condition (this study). Before the stress test was conducted as reported in this study, the RAS was operated under a normal condition for 10 weeks (3 weeks of RAS conditioning, 3 weeks of algae adaptation, and 4 weeks of experiment under normal condition comparing between RAS+A and RAS-A). During the experiment under a normal condition (without a stressor), TAN and NO_2_-N concentration below 1 mgL^-1^ were observed in both treatments. Meanwhile, NO_3_-N build-up was observed in both treatments though significantly lower NO_3_-N was observed in RAS+A than RAS-A (data not shown).

Therefore, the stress test was conducted to see the effects of algae inclusion on the RAS resistance and resilience towards the pH stressor. TAN, NO_2_-N and NO_3_-N were measured at three different points, fish tank, nitrification tank, and algae tank. The values were used to estimate the production of TAN in the fish tank, and to evaluate the performance of the nitrification and algae tanks on their role on converting or assimilating TAN, NO_2_-N or NO_3_-N. However, the results showed that there were no significant differences of TAN, NO_2_-N, and NO_3_-N between the sampling locations (S1 Table). This might be due to high flow rate (6 L min^-1^ for nitrification tank and 3 L min^-1^ for algae tank), thus low retention time in the tanks caused only small changes of TAN, NO_2_-N and NO_3_-N in the tanks. Therefore, this study presented an average of TAN, NO_2_-N and NO_3_-N from the three sampling locations.

**Fig 2:**
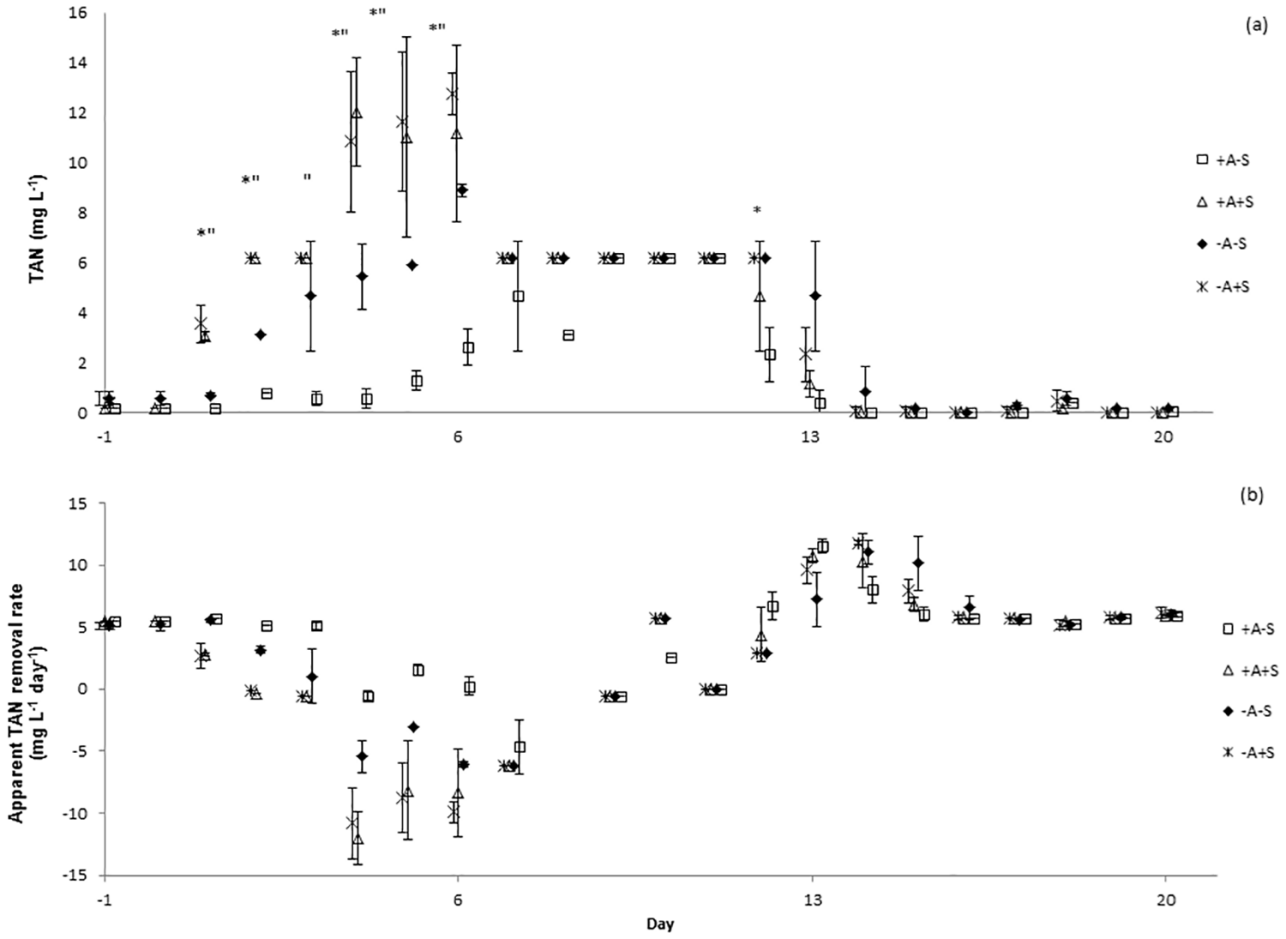
Means and standard deviation (SD) of total ammonia nitrogen (TAN) (mg L^-1^) in recirculating aquaculture systems (RAS). (a) (TAN) concentration (mg L^-1^). Points which are labeled with asterisk * show significant differences between algae and no-algae treatments and points which are labeled with asterisk “show significant differences between stressed and non-stressed treatments on each day, p < 0.05. (b) TAN conversion rate (mg L^-1^ day^-1^).

**Fig 3:**
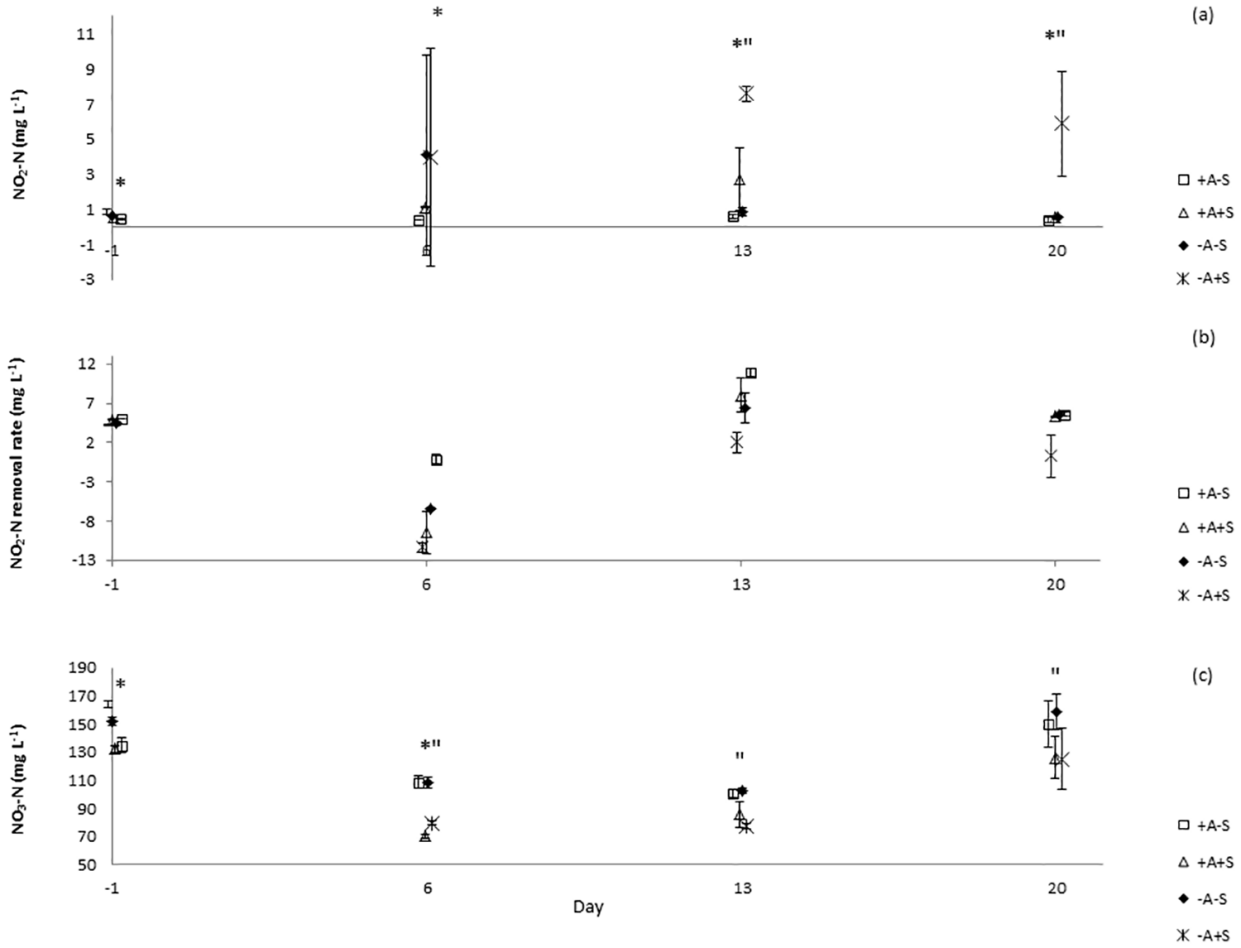
Means and standard deviation (SD) of nitrite-N (NO_2_-N) and nitrate (NO_3_-N) (mg L^-1^) in recirculating aquaculture systems (RAS). (a) NO_2_-N (mg L^-1^). (b) NO_2_-N conversion rate (mg L^-1^ day^-1^). (c) NO_3_-N (mg L^-1^). Points which are labeled with asterisk * show significant differences between algae and no-algae treatments and points which are labeled with asterisk “show significant differences between stressed and non-stressed treatments on each day, p < 0.05.

TAN concentrations increased in all systems immediately after the stressor was applied (Fig 2a). Water discharge was performed to control the level of TAN in stressed systems. However, the same water discharge procedure must also be done to control treatment (non-stressed RAS). Water discharge might cause bacterial wash-out and affected nitrifying bacteria. This might be the reason of TAN increased in control treatment after day 7. Unfortunately, from day 8 until day 11, instead of using phenate method, TAN was analyzed using API ammonia test kit due to technical problem with spectrophotometer. The kit could detect a maximum TAN level of 8 mg L^-1^. From the color indicator, the ranges of water quality in all treatments were more than 4, but below 8 mg L^-1^. Even though there were differences between treatments from day 1 onwards when the phenate method was used, the test kit was not sensitive enough to detect the differences. This was the reason of the same TAN level on day 8 until 11 as shown in Fig 2a. Significant differences (p < 0.05) of TAN concentrations were explained by the factors algae, stressor and day, but not by sample location (S1 Table). In RAS, ammonium may be removed via three processes; conversion to nitrite and subsequently to nitrate through nitrification, immobilization in bacterial and archaeal biomass, and uptake by algae [19]. Since the experiment did not distinguish which process had caused the reduction of TAN in the RAS, apparent TAN conversion is the term used to describe the process. Apparent TAN conversion rate (mg L^-1^ day^-1^) (Fig 2b) was significantly affected by the factor stressor (S2 Table). Meanwhile, significant differences (p < 0.05) of nitrite concentrations were explained by the factors algae, stressor and day, but not by sample location (S1 Table). Nitrite concentration was below 1 mg L^-1^ in all treatments on d-1. In RAS-A+S, nitrite increased after the stressor was applied and on d20 after stress, its concentration was 6 ± 3 mg L^-1^ (Fig 3a). However, for RAS+A+S, nitrite was below 1 mg L^-1^ during the experiment except on day 13 when the level was 3 ± 2 mg L^-1^. For RAS+A-S, NO_2_-N was below 1 mg L^-1^ throughout the experiment as a result of reduced TAN oxidation and dilution. Apparent nitrite conversion rate (Fig 3b) was significantly affected by the factor algae (S2 Table). Nitrate levels decreased in all systems on day 6 and 13 after the stress application (Fig 3c). Significant difference (p < 0.05) of nitrate was found between the factors algae, stressor, and day (S1 Table).

These results showed that lowering the pH in RAS from pH 7 to pH 4 and maintaining it for three hours disrupted the function of the bacterial communities in the RAS+A and RAS-A as indicated by the deteriorated water quality following the stress application. Similarly, a study on bacterial communities in lakes and rivers found that a low pH was unfavorable for bacterial growth [21] and in soilless cultivation media, low pH resulted in a significant decrease of ammonia oxidation rates and ammonia oxidizing bacteria community diversity [22].

Resistance towards the acute pH drop for RAS+A and RAS-A was not significantly different neither for apparent TAN nor for nitrite conversion (Table 1). The same result was found for the resilience for TAN conversion. However, the resilience for nitrite conversion was significantly higher for the RAS+A than for RAS-A. Therefore, we concluded that the efficiency of ammonium conversion was not different in both treatments. However, since the nitrite and nitrate concentrations were significantly lower in RAS+A than in RAS-A this might indicate that algae could have absorbed some ammonium, thus less ammonium was available for nitrification, subsequently less ammonium was converted to nitrite and nitrate. Additionally the resilience for nitrite conversion was significantly higher in the RAS+A than RAS-A, indicating that algae had a positive effect on RAS water quality.

**Table 1:** Resistance and resilience to an acute pH drop for total ammonia nitrogen (TAN) and nitrite-N conversion rate.

TAN conversion rate				Nitrite conversion rate			
Resistance		Resilience		Resistance		Resilience	
**+A**	-A	+A	-A	+A	-A	+A	-A
**-0.28a**	-0.30a	0.89a	0.80a	-0.27a	-0.32a	0.79a	0.57b

Means between recirculating aquaculture system with (+A) and without (–A) algae followed by different letter are statistically different by t-test (P < 0.05).

In this experiment, TAN production was expected to be similar in all systems which equal to 1475 mg TAN per day (40g feed per day X 40% protein X 0.092) which was equivalent to 5.67 mgL^-1^ TAN per day. This estimation was based on Timmons et al., (2002). The assimilation of ammonium by algae is normally estimated using the photosynthetic rate. However, since such data were not available the assimilation rate might be estimated using the stoichiometric relationship of phototrophic algal metabolism [19]. Algae chlorophyll-a content in this study was 5.8 mg per RAS+A (22.3 μg L^-1^). Considering that chlorophyll-a content was 1% from the algae dry weight, a total biomass of 580 mg dry weight algae was estimated to be present in the system. Every gram of ammonium nitrogen assimilated by algae will yield 15.58 g algal biomass [19]. Therefore, 580 mg algal biomass in the experiment might have assimilated 37 mg ammonium which was approximately 2.5% from the TAN produced by the RAS. When the microbial community was stressed uptake of ammonium by algae might stabilize the system and contribute to lower nitrite in RAS+A than RAS-A.

Effects of the algae on pH were mainly observed during the pH lowering from 7 to 4 where significantly more (P-value< 0.05) hydrochloric acid was added to RAS+A (85.5 ± 12.02 ml) than RAS-A (26.5 ± 0.71 ml). The presence of algae in RAS+A and uptake of CO_2_ during the photosynthesis could have contributed to the observed stability of pH in the RAS+A treatment. No pH diurnal effect was observed later throughout the study most probably because of water exchange which was conducted to control the level of TAN in the RAS. pH in RAS-A was 6.81 ±0.26 and in RAS+A was 6.87 ±0.29.

### Overall bacterial diversity

Miseq Illumina 16S rRNA gene fragments were used to profile the bacterial communities in RAS. Trimming and quality filtering of the raw reads generated 9,419,626 high-quality reads. Removal of chimeric sequences reduced the number to 9,080,633 reads for downstream analysis. Finally, 8,000,540 sequences were clustered into 5561 OTUs at a similarity threshold of 97% into the bacteria domains. The minimum read count per sample was 315,930 and the maximum was 580,980. Rarefaction curves showed leveling off in all bacterial communities for all samples at maximum sequence depth of 315,930 (S3 Fig).

Overall, 26 bacterial phyla were detected from which Proteobacteria (alpha, beta and gamma) covered 42% of the total sequences. The second most abundant phylum was Actinobacteria (21% of the total) which was dominated by the class Actinobacteria. The third most abundant phylum was Verrucomicrobia (10.6% of the total) which was dominated by the class Verrucomicrobiae. Other major phyla were Bacteroidetes (8.6%, represented by the classes Bacteroidia, Flavobacteriia and Cytophagia), Fusobacteria (6.1%, represented by the only class Fusobacteria), Planctomycetes (5.0%, mainly represented by the class Planctomycetia), Chloroflexi (2.5%), Nitrospirae (1.1%), Acidobacteria (0.5%) and Firmicutes (0.5%).

### Bacterial community structure in RAS with and without algae

#### Day -1 (before stressor)

**Fig 4:**
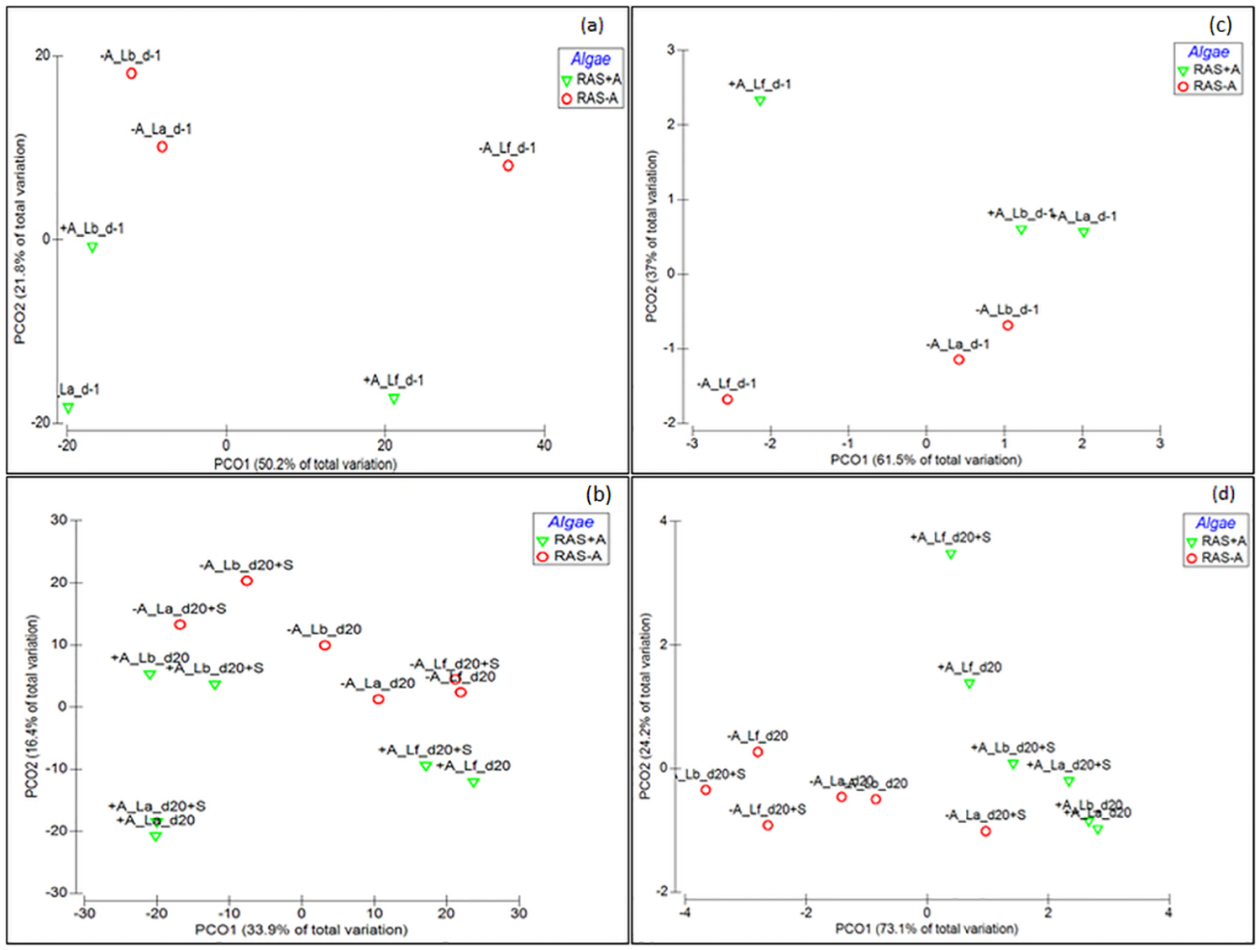
Bacterial communities in recirculating aquaculture systems (RAS) based on Bray-Curtis distance of relative abundance of operational taxonomic unit (OTU) data. (a) a day before stress (d-1). (b) 20 days after stress (d20); **Functional categories based on Bray-Curtis distance of relative abundance of cluster of orthologous genes (COG) data** (c) a day before stress (d-1). (d) 20 days after stress (d20). Samples are labeled with factors “algae”- with algae (+A), without algae (-A); “location”- fish (Lf), algae (La) and bio-filter (Lb) tanks; “day”- a day before stress (d-1).

Bacterial communities from RAS+A were clustered at the lower half of y-axis and RAS-A were clustered at the upper half of y-axis (Fig 4a). No difference was found between RAS+A and RAS-A (Pseudo-F = 3.9; P-value = 0.056; Unique permutations = 60), but a significant difference was found between fish, algae and nitrification tanks (Pseudo-F = 5.6; P-value = 0.03).

When predicted functions based on COG categories of bacterial community on d-1 were plotted, a separation can be seen (Fig 4c). A significant difference was found between RAS+A and RAS-A (Pseudo-F = 7.2; P-value = 0.049; Unique permutations = 60) and a significant difference was found between fish, algae and nitrification tanks (Pseudo-F = 7.1; P-value = 0.045). The results from d-1 showed that algae affected bacterial community in the RAS.

Summary of COG categories was plotted in S4 Fig. “Organismal systems” and “human disease” which were less relevant to environmental samples [23] were omitted in the diagram.

#### Day 20 (after stressor)

The results from this study strongly suggested that algae influenced the bacterial composition and functions in the RAS as the effect of algae was also observed on day 20 after stress. The ordination of the bacterial communities showed that bacterial communities from RAS+A were separated from the bacterial communities of RAS-A (Fig 4b). When bacterial communities were compared between factors algae, location and stressor, the results showed that there were significant differences (P < 0.05) of bacterial communities for all factors (S3 Table). A separation can also be seen when predicted functions based on COG categories of bacterial community were plotted (Fig 4d). A significant difference of predicted functions was found between RAS+A and RAS-A (S3 Table).

#### Discriminant OTUs—Algae effect

**Fig 5:**
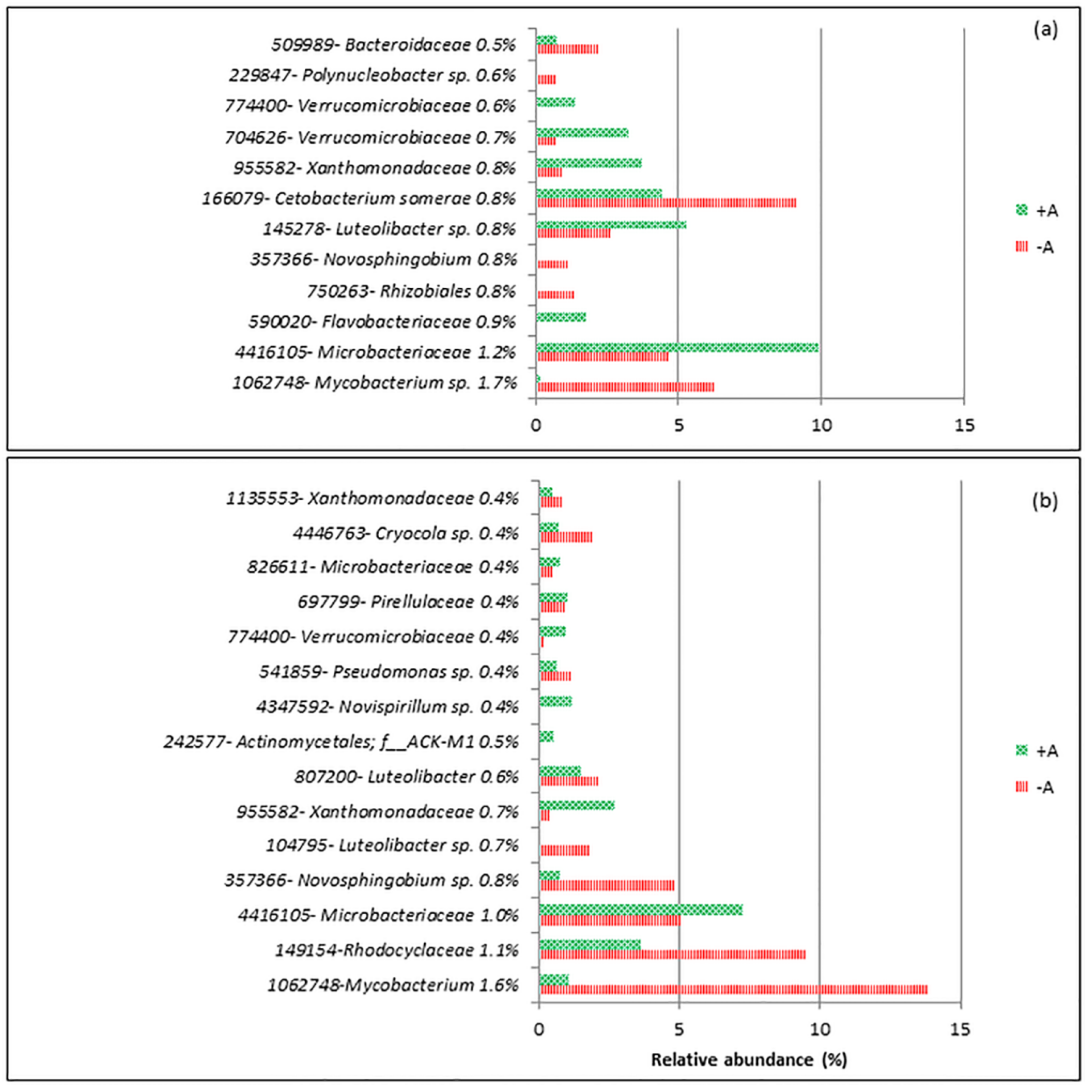
Operational taxonomy unit (OTU) dissimilarity between recirculating aquaculture system (RAS) with (+A) and without (-A) algae. (a) a day before stress (d-1). (b) 20 days after stress (d20). The graphs show abundances of the top 10% OTU that contributed to the total dissimilarity as given by SIMPER analysis. A number of percentage (%) written next to the identity of OTU denoted the % of contribution to the dissimilarity between the RAS.

SIMPER analysis showed that for d-1, the dissimilarity between RAS+A and RAS-A was 34% (Bray Curtis dissimilarity index). SIMPER listed 379 OTUs (6.8% of total OTUs) which represented 50% from the total 34% dissimilarity. In total, 5561 OTUs were obtained in this experiment. Here, only 12 OTUs were listed which contributed to the top 10% from the total 34% dissimilarity due to the algae factor (Fig 5a). The dissimilarity between RAS+A and RAS-A on d20 was 44%. SIMPER results listed 15 OTU that contributed to the top 10% from the total dissimilarity between the treatments (Fig 5b). *Mycobacterium* sp. and *Novosphingobium* sp. were the two groups that were consistently higher in RAS-A than RAS+A on d-1 and d20 after stress. Meanwhile, Microbacteriaceae, Xanthomonadaceae, and Verrucomicrobiaceae were found consistently higher in RAS+A than RAS-A on d-1 and d20 after stress.

**Fig 6:**
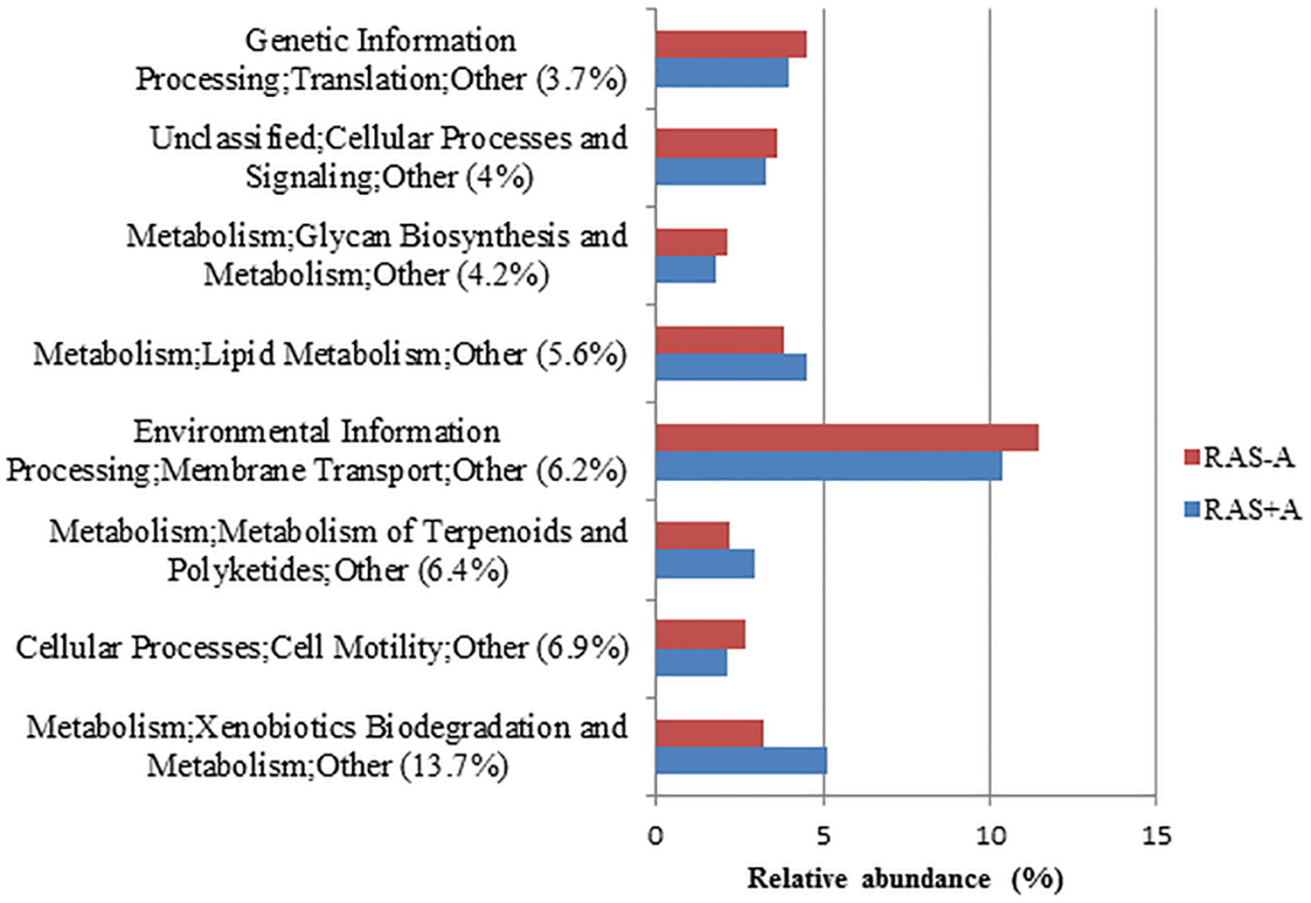
Predicted functions dissimilarity between recirculating aquaculture system (RAS) with (+A) and without (-A) algae. The predicted functions were based on cluster of orthologous genes (COG). The graph shows abundances of the top 50% COG that contributed to the total dissimilarity as given by SIMPER analysis. A number of percentage (%) written next to the functions denoted the % of contribution to the dissimilarity between the RAS.

Based on SIMPER analysis of COG categories, dissimilarity between RAS+A and RAS-A was 3.73%. Functional category “xenobiotic biodegradation and metabolism” was the highest discriminant (14%) from the total dissimilarity (Fig 6).

From the results, most of the discriminant bacteria that contributed to the differences between RAS+A and RAS–A were heterotrophic bacteria. This could mean that the different bacterial composition might be caused by the dynamics of organic nutrients in the system [24,25]. This is very plausible since xenobiotic degradation and metabolism was the highest discriminant function between RAS+A and RAS-A. Xenobiotic compounds are generally known as chemicals that are not natural to the environment and are regarded as environmental pollutants [26]. In the RAS-A, *Mycobacterium* sp. from the phylum Actinobacteria was more abundant than in RAS+A. This species is ubiquitous and has the ability to degrade polycyclic aromatic hydrocarbons (PAHs) which are environmental pollutants in all aquatic environments including tap water [27]. Therefore, this species is regarded as a potential bioremediation agent [27]. Furthermore, *Mycobacterium* is also versatile in using any carbon sources. *Novosphingobium* sp. which was also higher in the RAS-A is a genus within the alpha subclass of Proteobacteria. This genus is Gram-negative, non-sporulating, strictly aerobic, chemo-organotrophic and able to reduce nitrate [28]. This species is known to be metabolically versatile, often associated with biodegradation of aromatic compounds which is the reason the species is often regarded as a bioremediation agent [29–31]. Though some studies showed that algae had the ability to degrade xenobiotic compounds which might be the reason why these bacteria were less in the RAS+A, such conclusion cannot be made until a further test was conducted on the algae.

In the meanwhile, bacteria that were more dominant in the RAS+A had the ability to degrade organic nutrients originated by microalgae. For example, the family Verrucomicrobiaceae which was more abundant in RAS+A than in RAS-A, is member of the phylum Verrucomicrobia [32]. Some genera which were found under this family were Gram-negative, facultative anaerobic, non-motile and able to degrade algal metabolites as discovered for *Prosthecobacter algae* [33]. The other important group that was found more abundant in RAS+A was *Luteolibacter* sp. also a member of the family Verrucomicrobiaceae [34]. In the study of Park et al. [35], *Luteolibacter yonseiensis*, a Gram-negative aerobic and heterotrophic bacterium was isolated from activated sludge using algal metabolites. This could mean that *Luteolibacter* sp. and some other members under the family Verrucomicrobiaceae which were found in our study might be able to degrade algal metabolites in the RAS+A. Flavobacteriaceae was also higher in the RAS+A than RAS-A. This family is from the phylum Bacteroidetes which are normally regarded as specialists in the degradation of high-molecular weight organic matter which might be the reason why it is normally in association with algae [36]. It was also reported that Flavobacteria-Sphingobacteria group of the Bacteroidetes phylum were among the main bacteria group that were associated with diatoms [37]. Meanwhile, *Flavobacterium algicola* has been reported as having the ability to degrade fucoidan, a type of polysaccharide which originate from brown macroalgae [38]. Summarizing, our data showed that the presence of algae stimulates bacterial species which metabolize organic compound released by the algae.

#### Discriminant OTUs—Stress effect

On d20, the dissimilarity between stressed and non-stressed RAS, as given by Bray-Curtis index, was 43%. SIMPER listed 20 OTUs that contributed to the top 10% from the total dissimilarity between the +S and -S (S5 Fig). C39 sp., *Novosphingobium* sp., Xanthomonadaceae, Verrucomicrobiaceae, *Pseudomonas* sp., and *Cryocola* sp., were among the most discriminant in the non-stressed systems and Microbacteriaceae, *Mycobacterium* sp., *Luteolibacter* sp., Aeromonadaceae, Pirellulaceae, and *Nitrospira* sp. were among the most discriminant group in the stressed system. Twenty days after the stressor was applied, even though the bacterial communities between +S and–S were different, PICRUSt showed that there were no significant difference functions between +S and -S systems (S3 Table). The bacteria species those were more abundant in +S than in -S systems indicated that the stressor influenced the abundance of bioremedial species which contributed to maintaining system functionality. In addition, stressful system (+S system) usually provides room for tolerant species, such as the members of the genus *Mycobacterium* which are known to be tolerant to low pH [39].

#### Nitrifying bacteria

This study found Nitrosomonadaceae and *Nitrospira* as bacteria involved in autotrophic nitrification, whilst for heterotrophic nitrification and denitrification *Rhodococcus* [40], *Chryseobacterium* [41], *Bacillus* [42], *Acinetobacter* [43], and *Pseudomonas* [44] were the groups of bacteria involved (S6 Fig). Presence of Nitrosomonadaceae was almost negligible in all RAS (relative abundance < 0.05%). More changes of these bacteria occurred in RAS-A than RAS+A. However, the relative abundance of these bacteria was not significantly different between RAS-A and RAS+A (Pseudo-F = 0.8436; P-value = 0.475; Unique permutations = 974). These bacteria count about 3.5 to 10% from the total bacterial abundance and their presence was not affected by algae. Bacteria which were affected by the algae were mostly from the heterotrophic group. It was expected that the algae concentration was too low to reduce TAN availability for nitrification to measure effects. Therefore, in the future an experiment which will allow a higher immobilization of ammonium by algal biomass should be conducted to be able to measure algae effect on nitrifiers.

## Conclusion

The study showed that RAS with and without algae had the same resistance and resilience in restoring to pre-stressor maintenance of low ammonium levels after an acute pH perturbation. Algae supported RAS in keeping the nitrite concentration low before and after the perturbation. In this regard, this research concluded that RAS+A had a better stability than RAS-A. Algae influenced the bacterial community composition in the RAS causing more algal-associated bacteria species to be found in the RAS+A. This suggests strongly that algae can be used to manipulate the bacterial community in RAS.

## Supporting information

S1 Table Three way repeated measure analysis of variance of total ammonia nitrogen (TAN), nitrite (NO_2_-N), and nitrate (NO_3_-N) concentration (mg L^-1^) in recirculating aquaculture systems. The results compare between factors algae (with algae (+A) and without algae (-A)), location (fish, algae and nitrification), stressor (with stressor (+S) and without stressor (-S)) and day (-1,6,13 and 20).
(PDF)

S2 Table Repeated measure analysis of variance of apparent total ammonia nitrogen (TAN) conversion rate (mg L^-1^ day^-1^) and apparent nitrite (NO_2_-N) conversion rate (mg L^-1^ day^-1^) in recirculating aquaculture systems. The results compare between factors algae (with algae (+A) and without algae (-A)), and stressor (with stressor (+S) and without stressor (-S)) in recirculating aquaculture systems.
(PDF)

S3 Table Microbiota differences based on operational taxonomy units (OTU) and cluster of orthologous genes (COG). (PDF)

S4 Table Water quality data (min, max, mean, and standard deviation). (PDF)

S1 Fig pH changes in recirculating aquaculture system (RAS) with algae (+A) and without algae (-A). (TIF)

S2 Fig Percentage (%) of daily water replacement from recirculating aquaculture system on days after pH drop was applied. (TIF)

S3 Fig Curves based on Chao1 (richness analysis) at a sequencing depth of 315930. Samples are labeled with factors “algae”- with algae (+A), without algae (-A); “location”- fish (Lf), algae (La) and bio-filter (Lb) tanks; “day”- a day before stress (d-1), 20 days after stress (d20) and “stressor”- stressed (+S) and not stressed (-S).
(TIF)

S4 Fig Percentages of predicted sequences by cluster of orthologous genes (COG). Samples are labeled with factors “algae”- with algae (+A), without algae (-A); “location”- fish (Lf), algae (La) and bio-filter (Lb) tanks; and stressor- stressed (+S) and not stressed (-S).
(TIF)

S5 Fig Operational taxonomy unit (OTU) dissimilarity of bacterial community between stressed (+S) and non-stressed (-S) recirculating aquaculture system (RAS). The graph shows the top 10% OTU which contributed to the total dissimilarity as given by SIMPER analysis. A number of percentage (%) written next to the identity of OTU denoted the % of contribution to the dissimilarity between +S and -S.
(TIF)

S6 Fig Relative abundance of nitrifying bacteria. Bacteria which were able to perform autotrophic nitrification (Nitrosomonadaceae, Nitrospira) or heterotrophic nitrification and denitrification (Rhodococcus, Chryseobacterium, Bacillus, Acinetobacter, and Pseudomonas) identified in the recirculating aquaculture systems with (+A) and without algae (-A) a day before stress (d-1) and 20 days after stress (d20) which were stressed (+S) and not stressed (-S).
(TIF)
