# Supplementary material for: Resistance and resilience of small-scale recirculating aquaculture systems (RAS) with or without algae to pH perturbation
Source: PLoS One. 2018 Apr 16;13(4):e0195862. doi: 10.1371/journal.pone.0195862 (PMC5901992; doi:10.1371/journal.pone.0195862)
Supplement: S1 Table — The results compare between factors algae (with algae (+A) and without algae (-A)), location (fish, algae and nitrification), stressor (with stressor (+S) and without stressor (-S)) and day (-1,6,13 and 20). (PDF) [file pone.0195862.s001.pdf]

Parameters (mg L^{-1})	P-value								
	**Algae**	**Location**	**Stressor**	**Algae X Stressor**	**Day**	**Day X Algae**	**Day X Stressor**	**Day X AlgaeX Stressor**	**Day X Algae X Location**
**TAN**	<0.001	0.960	<0.001	0.002	<0.001	<0.001	<0.001	<0.001	1.000
**NO_2-N**	0.001	0.112	0.003	0.048	<0.001	0.001	<0.001	0.002	<0.001
**NO_3-N**	0.001	0.710	<0.001	0.456	<0.001	<0.001	<0.001	0.109	0.876
