# Supplementary material for: Resistance and resilience of small-scale recirculating aquaculture systems (RAS) with or without algae to pH perturbation
Source: PLoS One. 2018 Apr 16;13(4):e0195862. doi: 10.1371/journal.pone.0195862 (PMC5901992; doi:10.1371/journal.pone.0195862)
Supplement: S2 Table — The results compare between factors algae (with algae (+A) and without algae (-A)), and stressor (with stressor (+S) and without stressor (-S)) in recirculating aquaculture systems. (PDF) [file pone.0195862.s002.pdf]

	P-value						
**Parameters ($mg\ L^{-1}\ day^{-1}$)**	**Algae**	**Stressor**	**Algae X Stressor**	**Day**	**Day X Algae**	**Day X Stressor**	**Day X Algae X Stressor**
**Apparent TAN conversion rate**	0.062	0.001	0.061	<0.001	<0.001	<0.001	<0.001
**Apparent NO_2-N conversion rate**	<0.001	0.861	0.014	0.092	0.658	0.009	0.019
