# Supplementary material for: Resistance and resilience of small-scale recirculating aquaculture systems (RAS) with or without algae to pH perturbation
Source: PLoS One. 2018 Apr 16;13(4):e0195862. doi: 10.1371/journal.pone.0195862 (PMC5901992; doi:10.1371/journal.pone.0195862)
Supplement: S3 Table — (PDF) [file pone.0195862.s003.pdf]

| | | OTU based analysis | | COG based analysis | |
|---|---|---|---|---|---|
| **Source** | df | P(perm) | Unique perms | P(perm) | Unique perms |
| **Algae** | 1 | 0.002 | 999 | 0.012 | 998 |
| **Location** | 2 | 0.001 | 997 | 0.110 | 998 |
| **Stressor** | 1 | 0.005 | 998 | 0.664 | 999 |
| **Algae x Location** | 2 | 0.179 | 998 | 0.366 | 998 |
| **Algae x Stressor** | 1 | 0.01 | 996 | 0.430 | 998 |
| **Location x Stressor** | 2 | 0.37 | 999 | 0.567 | 999 |
| **Res** | 2 | | | | |
| **Total** | 11 | | | | |

RAS were compared between algae treatments (with algae, +A and without algae,-A), location (fish tank, bio-filters and algae tank) and stressor (when a stressor was applied, +S and when no stressor was applied, -S).
