# Supplementary material for: Resistance and resilience of small-scale recirculating aquaculture systems (RAS) with or without algae to pH perturbation
Source: PLoS One. 2018 Apr 16;13(4):e0195862. doi: 10.1371/journal.pone.0195862 (PMC5901992; doi:10.1371/journal.pone.0195862)
Supplement: S4 Table — (PDF) [file pone.0195862.s004.pdf]

Descriptive Statistics

	treatment	stress	Mean	Std. Deviation	N
d_1	RAS-A	no Stress	.5850	.27577	2
		Stress	.5850	.27577	2
		Total	.5850	.22517	4
	RAS+A	no Stress	.1900	.00000	2
		Stress	.1900	.00000	2
		Total	.1900	.00000	4
	Total	no Stress	.3875	.27813	4
		Stress	.3875	.27813	4
		Total	.3875	.25750	8
d0	RAS-A	no Stress	.5850	.27577	2
		Stress	.3900	.00000	2
		Total	.4875	.19500	4
	RAS+A	no Stress	.1900	.00000	2
		Stress	.1900	.00000	2
		Total	.1900	.00000	4
	Total	no Stress	.3875	.27813	4
		Stress	.2900	.11547	4
		Total	.3388	.20392	8
d1	RAS-A	no Stress	.7100	.09899	2
		Stress	3.5700	.73539	2
		Total	2.1400	1.70589	4
	RAS+A	no Stress	.1850	.00707	2
		Stress	3.0900	.15556	2
		Total	1.6375	1.67961	4
	Total	no Stress	.4475	.30848	4
		Stress	3.3300	.51491	4
		Total	1.8888	1.59008	8
d2	RAS-A	no Stress	3.1100	.00000	2
		Stress	6.2200	.00000	2
		Total	4.6650	1.79556	4
	RAS+A	no Stress	.7800	.00000	2
		Stress	6.2200	.00000	2
		Total	3.5000	3.14079	4
	Total	no Stress	1.9450	1.34523	4
		Stress	6.2200	.00000	4
		Total	4.0825	2.44891	8

		no Stress	4.6650	2.19910	2
	RAS-A	Stress	6.2200	.00000	2
		Total	5.4425	1.55500	4
		no Stress	.5850	.27577	2
d3	RAS+A	Stress	6.2200	.00000	2
		Total	3.4025	3.25726	4
		no Stress	2.6250	2.68070	4
	Total	Stress	6.2200	.00000	4
		Total	4.4225	2.60238	8
		no Stress	5.4500	1.31522	2
	RAS-A	Stress	10.8400	2.82843	2
		Total	8.1450	3.59546	4
		no Stress	.5850	.38891	2
d4	RAS+A	Stress	12.0350	2.15668	2
		Total	6.3100	6.73065	4
		no Stress	3.0175	2.91829	4
	Total	Stress	11.4375	2.16635	4
		Total	7.2275	5.09091	8
		no Stress	5.9100	.01414	2
	RAS-A	Stress	11.6450	2.79307	2
		Total	8.7775	3.68292	4
		no Stress	1.3050	.40305	2
d5	RAS+A	Stress	11.0250	3.99515	2
		Total	6.1650	6.07185	4
		no Stress	3.6075	2.66887	4
	Total	Stress	11.3350	2.83707	4
		Total	7.4713	4.85422	8
		no Stress	8.9050	.23335	2
	RAS-A	Stress	12.7600	.82024	2
		Total	10.8325	2.27949	4
		no Stress	2.6150	.72832	2
d6	RAS+A	Stress	11.1900	3.53553	2
		Total	6.9025	5.37156	4
		no Stress	5.7600	3.65828	4
	Total	Stress	11.9750	2.28310	4
		Total	8.8675	4.35954	8
		no Stress	6.2000	.00000	2
	RAS-A	Stress	6.2000	.00000	2
d7		Total	6.2000	.00000	4
	RAS+A	no Stress	4.6500	2.19203	2

		Stress	6.2000	.00000	2
		Total	5.4250	1.55000	4
		no Stress	5.4250	1.55000	4
	Total	Stress	6.2000	.00000	4
		Total	5.8125	1.09602	8
		no Stress	4.6650	2.19910	2
	RAS-A	Stress	6.2200	.00000	2
		Total	5.4425	1.55500	4
		no Stress	3.1100	.00000	2
d8	RAS+A	Stress	3.1100	.00000	2
		Total	3.1100	.00000	4
		no Stress	3.8875	1.55500	4
	Total	Stress	4.6650	1.79556	4
		Total	4.2763	1.60958	8
		no Stress	6.2200	.00000	2
	RAS-A	Stress	6.2200	.00000	2
		Total	6.2200	.00000	4
		no Stress	3.1100	.00000	2
d9	RAS+A	Stress	3.1100	.00000	2
		Total	3.1100	.00000	4
		no Stress	4.6650	1.79556	4
	Total	Stress	4.6650	1.79556	4
		Total	4.6650	1.66236	8
		no Stress	6.2000	.00000	2
	RAS-A	Stress	6.2000	.00000	2
		Total	6.2000	.00000	4
		no Stress	6.2000	.00000	2
d10	RAS+A	Stress	6.2000	.00000	2
		Total	6.2000	.00000	4
		no Stress	6.2000	.00000	4
	Total	Stress	6.2000	.00000	4
		Total	6.2000	.00000	8
		no Stress	6.2000	.00000	2
	RAS-A	Stress	6.2000	.00000	2
		Total	6.2000	.00000	4
d11		no Stress	6.2000	.00000	2
	RAS+A	Stress	6.2000	.00000	2
		Total	6.2000	.00000	4
	Total	no Stress	6.2000	.00000	4
		Stress	6.2000	.00000	4

		Total	6.2000	.00000	8
		no Stress	6.2000	.00000	2
	RAS-A	Stress	6.2000	.00000	2
		Total	6.2000	.00000	4
		no Stress	2.3500	1.06066	2
d12	RAS+A	Stress	4.6500	2.19203	2
		Total	3.5000	1.93391	4
		no Stress	4.2750	2.30561	4
	Total	Stress	5.4250	1.55000	4
		Total	4.8500	1.91982	8
		no Stress	3.8950	1.40714	2
	RAS-A	Stress	2.1350	.21920	2
		Total	3.0150	1.30712	4
		no Stress	.5200	.00000	2
d13	RAS+A	Stress	1.2200	.69296	2
		Total	.8700	.56868	4
		no Stress	2.2075	2.11114	4
	Total	Stress	1.6775	.67465	4
		Total	1.9425	1.47832	8
		no Stress	.9000	.98995	2
	RAS-A	Stress	.1000	.14142	2
		Total	.5000	.73937	4
		no Stress	.0000	.00000	2
d14	RAS+A	Stress	.0000	.00000	2
		Total	.0000	.00000	4
		no Stress	.4500	.77244	4
	Total	Stress	.0500	.10000	4
		Total	.2500	.55291	8
		no Stress	.1900	.00000	2
	RAS-A	Stress	.0950	.13435	2
		Total	.1425	.09500	4
		no Stress	.0000	.00000	2
d15	RAS+A	Stress	.0000	.00000	2
		Total	.0000	.00000	4
		no Stress	.0950	.10970	4
	Total	Stress	.0475	.09500	4
		Total	.0713	.09833	8
		no Stress	.0000	.00000	2
d16	RAS-A	Stress	.0000	.00000	2
		Total	.0000	.00000	4

		no Stress	.0000	.00000	2
	RAS+A	Stress	.0000	.00000	2
		Total	.0000	.00000	4
		no Stress	.0000	.00000	4
	Total	Stress	.0000	.00000	4
		Total	.0000	.00000	8
d17	RAS-A	no Stress	.2900	.14142	2
		Stress	.0950	.13435	2
		Total	.1925	.15924	4
	RAS+A	no Stress	.0000	.00000	2
		Stress	.0000	.00000	2
		Total	.0000	.00000	4
	Total	no Stress	.1450	.18628	4
		Stress	.0475	.09500	4
		Total	.0963	.14648	8
d18	RAS-A	no Stress	.6000	.28284	2
		Stress	.5000	.42426	2
		Total	.5500	.30000	4
	RAS+A	no Stress	.4000	.00000	2
		Stress	.2000	.00000	2
		Total	.3000	.11547	4
	Total	no Stress	.5000	.20000	4
		Stress	.3500	.30000	4
		Total	.4250	.24928	8
d19	RAS-A	no Stress	.2000	.00000	2
		Stress	.0000	.00000	2
		Total	.1000	.11547	4
	RAS+A	no Stress	.0000	.00000	2
		Stress	.0000	.00000	2
		Total	.0000	.00000	4
	Total	no Stress	.1000	.11547	4
		Stress	.0000	.00000	4
		Total	.0500	.09258	8
d20	RAS-A	no Stress	.2000	.00000	2
		Stress	.0000	.00000	2
		Total	.1000	.11547	4
	RAS+A	no Stress	.1000	.14142	2
		Stress	.0000	.00000	2
		Total	.0500	.10000	4

	no Stress	.1500	.10000	4
Total	Stress	.0000	.00000	4
	Total	.0750	.10351	8

Descriptive Statistics

	treatment	stress	tank	Mean	Std. Deviation	N
TAN.1.00	RAS without algae	No stress	Fish	.3300	.04243	2
			Algae	.3400	.05657	2
			Nitrification	.3650	.00707	2
			Total	.3450	.03564	6
		Stress	Fish	.4700	.08485	2
			Algae	.2900	.18385	2
			Nitrification	.4150	.09192	2
			Total	.3917	.12922	6
		Total	Fish	.4000	.09764	4
			Algae	.3150	.11475	4
			Nitrification	.3900	.06055	4
			Total	.3683	.09360	12
	RAS with algae	No stress	Fish	.1850	.06364	2
			Algae	.3500	.00000	2
			Nitrification	.5650	.31820	2
			Total	.3667	.22385	6
		Stress	Fish	.2500	.12728	2
			Algae	.1850	.14849	2
			Nitrification	.4550	.53033	2
			Total	.2967	.28247	6
		Total	Fish	.2175	.09032	4
			Algae	.2675	.12816	4
			Nitrification	.5100	.36268	4
			Total	.3317	.24572	12
	Total	No stress	Fish	.2575	.09465	4
			Algae	.3450	.03317	4
			Nitrification	.4650	.21703	4

			Total	.3558	.15324	12
		Stress	Fish	.3600	.15470	4
			Algae	.2375	.14930	4
			Nitrification	.4350	.31161	4
			Total	.3442	.21521	12
		Total	Fish	.3087	.13076	8
			Algae	.2913	.11544	8
			Nitrification	.4500	.24911	8
			Total	.3500	.18280	24
TAN.2.00	RAS without algae	No stress	Fish	8.9900	.22627	2
			Algae	8.9300	.22627	2
			Nitrification	8.7950	.26163	2
			Total	8.9050	.20530	6
		Stress	Fish	13.0300	.74953	2
			Algae	12.7200	.38184	2
			Nitrification	12.5150	1.33643	2
			Total	12.7550	.74331	6
		Total	Fish	11.0100	2.37589	4
			Algae	10.8250	2.20311	4
			Nitrification	10.6550	2.28713	4
			Total	10.8300	2.07673	12
	RAS with algae	No stress	Fish	2.6950	.53033	2
			Algae	2.5700	.83439	2
			Nitrification	2.5750	.81317	2
			Total	2.6133	.57597	6
		Stress	Fish	11.6400	2.98399	2
			Algae	10.6600	4.38406	2
			Nitrification	11.2750	3.23148	2
			Total	11.1917	2.81240	6
		Total	Fish	7.1675	5.45278	4
			Algae	6.6150	5.33430	4
			Nitrification	6.9250	5.37878	4
			Total	6.9025	4.88011	12
	Total	No stress	Fish	5.8425	3.64963	4
			Algae	5.7500	3.70572	4
			Nitrification	5.6850	3.62483	4
			Total	5.7592	3.31148	12
		Stress	Fish	12.3350	1.94920	4
			Algae	11.6900	2.80532	4

			Nitrification	11.8950	2.14213	4
			Total	11.9733	2.12437	12
		Total	Fish	9.0888	4.40232	8
			Algae	8.7200	4.39762	8
			Nitrification	8.7900	4.31463	8
			Total	8.8663	4.18051	24
TAN.3.00	RAS without algae	No stress	Fish	3.9000	1.45664	2
			Algae	3.9500	1.28693	2
			Nitrification	3.8400	1.47078	2
			Total	3.8967	1.09118	6
		Stress	Fish	2.1600	.25456	2
			Algae	2.1400	.16971	2
			Nitrification	2.1150	.23335	2
			Total	2.1383	.17325	6
		Total	Fish	3.0300	1.31836	4
			Algae	3.0450	1.28596	4
			Nitrification	2.9775	1.31571	4
			Total	3.0175	1.18239	12
	RAS with algae	No stress	Fish	.6000	.00000	2
			Algae	.4600	.00000	2
			Nitrification	.5000	.00000	2
			Total	.5200	.06450	6
		Stress	Fish	1.2300	.76368	2
			Algae	1.1750	.60104	2
			Nitrification	1.2500	.73539	2
			Total	1.2183	.54613	6
		Total	Fish	.9150	.57158	4
			Algae	.8175	.53928	4
			Nitrification	.8750	.60644	4
			Total	.8692	.52006	12
	Total	No stress	Fish	2.2500	2.08261	4
			Algae	2.2050	2.14758	4
			Nitrification	2.1700	2.10704	4
			Total	2.2083	1.91121	12
		Stress	Fish	1.6950	.71014	4
			Algae	1.6575	.66365	4
			Nitrification	1.6825	.66920	4
			Total	1.6783	.61648	12
		Total	Fish	1.9725	1.47070	8
			Algae	1.9313	1.50034	8

			Nitrification	1.9263	1.47055	8
			Total	1.9433	1.41492	24
TAN.4.00	RAS without algae	No stress	Fish	.4950	.14849	2
			Algae	.5800	.08485	2
			Nitrification	.3800	.05657	2
			Total	.4850	.12062	6
		Stress	Fish	.3350	.12021	2
			Algae	.3900	.11314	2
			Nitrification	.1950	.00707	2
			Total	.3067	.11639	6
		Total	Fish	.4150	.14387	4
			Algae	.4850	.13675	4
			Nitrification	.2875	.11177	4
			Total	.3958	.14644	12
	RAS with algae	No stress	Fish	.2800	.08485	2
			Algae	.3500	.11314	2
			Nitrification	.4500	.22627	2
			Total	.3600	.14170	6
		Stress	Fish	.2350	.04950	2
			Algae	.3400	.07071	2
			Nitrification	.2300	.00000	2
			Total	.2683	.06765	6
		Total	Fish	.2575	.06238	4
			Algae	.3450	.07724	4
			Nitrification	.3400	.18221	4
			Total	.3142	.11619	12
	Total	No stress	Fish	.3875	.15861	4
			Algae	.4650	.15588	4
			Nitrification	.4150	.14059	4
			Total	.4225	.14143	12
		Stress	Fish	.2850	.09469	4
			Algae	.3650	.08226	4
			Nitrification	.2125	.02062	4
			Total	.2875	.09294	12
		Total	Fish	.3363	.13277	8
			Algae	.4150	.12717	8
			Nitrification	.3138	.14272	8
			Total	.3550	.13584	24
TAN.5.00	RAS without algae	No stress	Fish	.9800	.19799	2
			Algae	.9800	.19799	2

		Nitrification	.9800	.19799	2
		Total	.9800	.15336	6
	Stress	Fish	.1250	.03536	2
		Algae	.1250	.03536	2
		Nitrification	.1250	.03536	2
		Total	.1250	.02739	6
	Total	Fish	.5525	.50711	4
		Algae	.5525	.50711	4
		Nitrification	.5525	.50711	4
		Total	.5525	.45870	12
RAS with algae	No stress	Fish	.0900	.09899	2
		Algae	.0900	.09899	2
		Nitrification	.0900	.09899	2
		Total	.0900	.07668	6
	Stress	Fish	.2850	.04950	2
		Algae	.2850	.04950	2
		Nitrification	.2850	.04950	2
		Total	.2850	.03834	6
	Total	Fish	.1875	.12945	4
		Algae	.1875	.12945	4
		Nitrification	.1875	.12945	4
		Total	.1875	.11710	12
Total	No stress	Fish	.5350	.52950	4
		Algae	.5350	.52950	4
		Nitrification	.5350	.52950	4
		Total	.5350	.47895	12
	Stress	Fish	.2050	.09883	4
		Algae	.2050	.09883	4
		Nitrification	.2050	.09883	4
		Total	.2050	.08939	12
	Total	Fish	.3700	.39428	8
		Algae	.3700	.39428	8
		Nitrification	.3700	.39428	8
		Total	.3700	.37675	24

Descriptive Statistics

	treatment	stress	tank	Mean	Std. Deviation	N
NO2.1.00	RAS without algae	No stress	Fish	.6600	.01414	2
			Algae	.5800	.04243	2
			Nitrification	.6300	.01414	2
			Total	.6233	.04179	6
		Stress	Fish	.8550	.19092	2
			Algae	.8200	.21213	2
			Nitrification	.8250	.16263	2
			Total	.8333	.14787	6
		Total	Fish	.7575	.15777	4
			Algae	.7000	.18655	4
			Nitrification	.7275	.14683	4
			Total	.7283	.15087	12
	RAS with algae	No stress	Fish	.4650	.03536	2
			Algae	.3750	.00707	2
			Nitrification	.4400	.05657	2
			Total	.4267	.05125	6
		Stress	Fish	.6050	.03536	2
			Algae	.5300	.02828	2
			Nitrification	.5450	.00707	2
			Total	.5600	.04099	6
		Total	Fish	.5350	.08583	4
			Algae	.4525	.09106	4
			Nitrification	.4925	.06898	4
			Total	.4933	.08250	12
	Total	No stress	Fish	.5625	.11471	4
			Algae	.4775	.12093	4
			Nitrification	.5350	.11475	4
			Total	.5250	.11197	12
		Stress	Fish	.7300	.18276	4
			Algae	.6750	.20809	4
			Nitrification	.6850	.18699	4
			Total	.6967	.17629	12
		Total	Fish	.6463	.16724	8
			Algae	.5762	.18966	8

			Nitrification	.6100	.16449	8
			Total	.6108	.16896	24
	RAS without algae	No stress	Fish	11.4800	.29698	2
			Algae	.3900	.00000	2
			Nitrification	.3950	.00707	2
			Total	4.0883	5.72710	6
		Stress	Fish	9.0600	10.80459	2
			Algae	1.3900	.01414	2
			Nitrification	1.4100	.01414	2
			Total	3.9533	6.24458	6
		Total	Fish	10.2700	6.39489	4
			Algae	.8900	.57741	4
			Nitrification	.9025	.58608	4
			Total	4.0208	5.71304	12
NO2.2.00	RAS with algae	No stress	Fish	.3750	.00707	2
			Algae	.3600	.01414	2
			Nitrification	.3650	.00707	2
			Total	.3667	.01033	6
		Stress	Fish	1.1100	.05657	2
			Algae	1.0800	.00000	2
			Nitrification	1.1300	.05657	2
			Total	1.1067	.04227	6
		Total	Fish	.7425	.42563	4
			Algae	.7200	.41577	4
			Nitrification	.7475	.44290	4
			Total	.7367	.38756	12
	Total	No stress	Fish	5.9275	6.41377	4
			Algae	.3750	.01915	4
			Nitrification	.3800	.01826	4
			Total	2.2275	4.32279	12
		Stress	Fish	5.0850	7.74478	4
			Algae	1.2350	.17916	4
			Nitrification	1.2700	.16513	4
			Total	2.5300	4.46495	12
		Total	Fish	5.5063	6.59842	8
			Algae	.8050	.47458	8
			Nitrification	.8250	.48800	8
			Total	2.3787	4.30063	24
NO2.3.00	RAS without algae	No stress	Fish	.8450	.28991	2
			Algae	.8500	.25456	2

			Nitrification	.8300	.28284	2
			Total	.8417	.21414	6
		Stress	Fish	7.5950	.47376	2
			Algae	7.5850	.55861	2
			Nitrification	7.5700	.55154	2
			Total	7.5833	.41020	6
		Total	Fish	4.2200	3.91029	4
			Algae	4.2175	3.90457	4
			Nitrification	4.2000	3.90776	4
			Total	4.2125	3.53452	12
	RAS with algae	No stress	Fish	.6000	.12728	2
			Algae	.6000	.14142	2
			Nitrification	.5850	.13435	2
			Total	.5950	.10445	6
		Stress	Fish	2.6750	2.26981	2
			Algae	2.7400	2.36174	2
			Nitrification	2.6650	2.31224	2
			Total	2.6933	1.79348	6
		Total	Fish	1.6375	1.77706	4
			Algae	1.6700	1.84186	4
			Nitrification	1.6250	1.79730	4
			Total	1.6442	1.63336	12
	Total	No stress	Fish	.7225	.23114	4
			Algae	.7250	.22159	4
			Nitrification	.7075	.22955	4
			Total	.7183	.20591	12
		Stress	Fish	5.1350	3.14022	4
			Algae	5.1625	3.12857	4
			Nitrification	5.1175	3.14694	4
			Total	5.1383	2.83902	12
		Total	Fish	2.9288	3.13240	8
			Algae	2.9437	3.13720	8
			Nitrification	2.9125	3.13423	8
			Total	2.9283	2.99525	24
NO2.4.00	RAS without algae	No stress	Fish	.5850	.00707	2
			Algae	.5300	.04243	2
			Nitrification	.5500	.00000	2
			Total	.5550	.03146	6
		Stress	Fish	6.4650	4.46184	2
			Algae	5.5650	3.47189	2

			Nitrification	5.6100	3.40825	2
			Total	5.8800	2.98687	6
		Total	Fish	3.5250	4.26155	4
			Algae	3.0475	3.53115	4
			Nitrification	3.0800	3.52230	4
			Total	3.2175	3.43351	12
	RAS with algae	No stress	Fish	.4600	.02828	2
			Algae	.4400	.00000	2
			Nitrification	.2250	.30406	2
			Total	.3750	.17953	6
		Stress	Fish	.5800	.01414	2
			Algae	.5300	.08485	2
			Nitrification	.5550	.03536	2
			Total	.5550	.04722	6
		Total	Fish	.5200	.07165	4
			Algae	.4850	.07141	4
			Nitrification	.3900	.25987	4
			Total	.4650	.15652	12
	Total	No stress	Fish	.5225	.07411	4
			Algae	.4850	.05745	4
			Nitrification	.3875	.25695	4
			Total	.4650	.15471	12
		Stress	Fish	3.5225	4.26386	4
			Algae	3.0475	3.53141	4
			Nitrification	3.0825	3.51997	4
			Total	3.2175	3.43359	12
		Total	Fish	2.0225	3.21954	8
			Algae	1.7662	2.68741	8
			Nitrification	1.7350	2.72278	8
			Total	1.8412	2.76158	24
NO2.5.00	RAS without algae	No stress	Fish	.3700	.00000	2
			Algae	.3700	.00000	2
			Nitrification	.3700	.00000	2
			Total	.3700	.00000	6
		Stress	Fish	.4500	.00000	2
			Algae	.4500	.00000	2
			Nitrification	.4500	.00000	2
			Total	.4500	.00000	6
		Total	Fish	.4100	.04619	4

				Mean	Std. Deviation	N
			Algae	.4100	.04619	4
			Nitrification	.4100	.04619	4
			Total	.4100	.04178	12
	RAS with algae	No stress	Fish	.3950	.02121	2
			Algae	.3950	.02121	2
			Nitrification	.3950	.02121	2
			Total	.3950	.01643	6
		Stress	Fish	.4050	.02121	2
			Algae	.4050	.02121	2
			Nitrification	.4050	.02121	2
			Total	.4050	.01643	6
		Total	Fish	.4000	.01826	4
			Algae	.4000	.01826	4
			Nitrification	.4000	.01826	4
			Total	.4000	.01651	12
	Total	No stress	Fish	.3825	.01893	4
			Algae	.3825	.01893	4
			Nitrification	.3825	.01893	4
			Total	.3825	.01712	12
		Stress	Fish	.4275	.02872	4
			Algae	.4275	.02872	4
			Nitrification	.4275	.02872	4
			Total	.4275	.02598	12
		Total	Fish	.4050	.03295	8
			Algae	.4050	.03295	8
			Nitrification	.4050	.03295	8
			Total	.4050	.03148	24

Descriptive Statistics

	treatment	stress	tank	Mean	Std. Deviation	N
NO3.1.00	RAS without algae	No stress	Fish	151.4700	3.57796	2
			Algae	151.7650	4.58912	2
			Nitrification	153.4700	2.24860	2
			Total	152.2350	2.95231	6
		Stress	Fish	164.1800	1.96576	2

			Algae	162.0900	1.14551	2
			Nitrification	166.2750	3.37290	2
			Total	164.1817	2.61025	6
		Total	Fish	157.8250	7.70736	4
			Algae	156.9275	6.55688	4
			Nitrification	159.8725	7.75458	4
			Total	158.2083	6.78110	12
	RAS with algae	No stress	Fish	134.1650	6.79530	2
			Algae	135.5400	8.04688	2
			Nitrification	134.9650	6.54074	2
			Total	134.8900	5.57884	6
		Stress	Fish	132.1550	2.15668	2
			Algae	133.2150	.12021	2
			Nitrification	131.8300	4.70933	2
			Total	132.4000	2.40590	6
		Total	Fish	133.1600	4.27658	4
			Algae	134.3775	4.83640	4
			Nitrification	133.3975	4.99290	4
			Total	133.6450	4.29756	12
	Total	No stress	Fish	142.8175	10.93070	4
			Algae	143.6525	10.78676	4
			Nitrification	144.2175	11.40574	4
			Total	143.5625	10.00793	12
		Stress	Fish	148.1675	18.56624	4
			Algae	147.6525	16.68425	4
			Nitrification	149.0525	20.16608	4
			Total	148.2908	16.76911	12
		Total	Fish	145.4925	14.39148	8
			Algae	145.6525	13.18091	8
			Nitrification	146.6350	15.38570	8
			Total	145.9267	13.71942	24
NO3.2.00	RAS without algae	No stress	Fish	108.6850	4.09415	2
			Algae	107.6550	3.01935	2
			Nitrification	108.6050	5.76292	2
			Total	108.3150	3.47571	6
		Stress	Fish	80.5500	.43841	2

			Algae	78.1350	1.27986	2
			Nitrification	80.3350	.28991	2
			Total	79.6733	1.34610	6
		Total	Fish	94.6175	16.41678	4
			Algae	92.8950	17.14823	4
			Nitrification	94.4700	16.65821	4
			Total	93.9942	15.16724	12
	RAS with algae	No stress	Fish	109.7750	6.54074	2
			Algae	107.3400	3.80423	2
			Nitrification	108.9250	6.61145	2
			Total	108.6800	4.62761	6
		Stress	Fish	71.7600	.97581	2
			Algae	68.9900	.00000	2
			Nitrification	69.6600	2.40416	2
			Total	70.1367	1.73705	6
		Total	Fish	90.7675	22.27759	4
			Algae	88.1650	22.25005	4
			Nitrification	89.2925	23.03064	4
			Total	89.4083	20.40260	12
	Total	No stress	Fish	109.2300	4.49931	4
			Algae	107.4975	2.80997	4
			Nitrification	108.7650	5.06705	4
			Total	108.4975	3.90660	12
		Stress	Fish	76.1550	5.11235	4
			Algae	73.5625	5.33132	4
			Nitrification	74.9975	6.31980	4
			Total	74.9050	5.19607	12
		Total	Fish	92.6925	18.23282	8
			Algae	90.5300	18.56312	8
			Nitrification	91.8813	18.81235	8
			Total	91.7013	17.73673	24
NO3.3.00	RAS without algae	No stress	Fish	101.4450	1.06773	2
			Algae	103.5400	4.31335	2
			Nitrification	102.4950	.07778	2
			Total	102.4933	2.19728	6
		Stress	Fish	77.4850	2.84964	2
			Algae	76.8600	2.14960	2
			Nitrification	76.8950	2.10011	2
			Total	77.0800	1.87856	6
		Total	Fish	89.4650	13.94444	4

			Algae	90.2000	15.65299	4
			Nitrification	89.6950	14.82989	4
			Total	89.7867	13.41403	12
	RAS with algae	No stress	Fish	100.6850	4.22143	2
			Algae	100.5150	3.59917	2
			Nitrification	101.2450	3.20319	2
			Total	100.8150	2.88508	6
		Stress	Fish	83.0650	9.05804	2
			Algae	86.3150	12.36730	2
			Nitrification	87.5950	12.40972	2
			Total	85.6583	9.06437	6
		Total	Fish	91.8750	11.69520	4
			Algae	93.4150	11.06864	4
			Nitrification	94.4200	10.81025	4
			Total	93.2367	10.18736	12
	Total	No stress	Fish	101.0650	2.55200	4
			Algae	102.0275	3.68373	4
			Nitrification	101.8700	1.98570	4
			Total	101.6542	2.59736	12
		Stress	Fish	80.2750	6.35885	4
			Algae	81.5875	9.07318	4
			Nitrification	82.2450	9.53767	4
			Total	81.3692	7.68246	12
		Total	Fish	90.6700	11.98387	8
			Algae	91.8075	12.66753	8
			Nitrification	92.0575	12.27664	8
			Total	91.5117	11.78119	24
NO3.4.00	RAS without algae	No stress	Fish	162.9150	1.81726	2
			Algae	162.5900	1.24451	2
			Nitrification	150.0000	24.23962	2
			Total	158.5017	12.72282	6
		Stress	Fish	130.3950	14.84217	2
			Algae	122.0700	32.03194	2
			Nitrification	122.0700	32.03194	2
			Total	124.8450	21.74759	6
		Total	Fish	146.6550	20.66513	4
			Algae	142.3300	29.82987	4
			Nitrification	136.0350	28.24706	4
			Total	141.6733	24.44372	12
	RAS with algae	No stress	Fish	150.4200	1.92333	2

			Algae	146.3350	34.18861	2
			Nitrification	153.6750	11.30664	2
			Total	150.1433	16.45907	6
		Stress	Fish	130.7000	4.03051	2
			Algae	113.3700	20.81722	2
			Nitrification	133.9650	12.14102	2
			Total	126.0117	14.74518	6
		Total	Fish	140.5600	11.67365	4
			Algae	129.8525	29.93832	4
			Nitrification	143.8200	14.87423	4
			Total	138.0775	19.51368	12
	Total	No stress	Fish	156.6675	7.37398	4
			Algae	154.4625	21.86805	4
			Nitrification	151.8375	15.58744	4
			Total	154.3225	14.68903	12
		Stress	Fish	130.5475	8.88122	4
			Algae	117.7200	22.62073	4
			Nitrification	128.0175	20.93594	4
			Total	125.4283	17.72510	12
		Total	Fish	143.6075	15.87569	8
			Algae	136.0913	28.45987	8
			Nitrification	139.9275	21.30939	8
			Total	139.8754	21.70820	24
NO3.5.00	RAS without algae	No stress	Fish	161.9800	3.69110	2
			Algae	161.9800	3.69110	2
			Nitrification	161.9800	3.69110	2
			Total	161.9800	2.85911	6
		Stress	Fish	163.7700	7.26906	2
			Algae	163.7700	7.26906	2
			Nitrification	163.7700	7.26906	2
			Total	163.7700	5.63059	6
		Total	Fish	162.8750	4.81897	4
			Algae	162.8750	4.81897	4
			Nitrification	162.8750	4.81897	4
			Total	162.8750	4.35893	12
	RAS with algae	No stress	Fish	158.8750	2.55266	2
			Algae	158.8750	2.55266	2
			Nitrification	158.8750	2.55266	2

		Total	158.8750	1.97728	6
	Stress	Fish	152.0850	4.40528	2
		Algae	152.0850	4.40528	2
		Nitrification	152.0850	4.40528	2
		Total	152.0850	3.41231	6
	Total	Fish	155.4800	4.89988	4
		Algae	155.4800	4.89988	4
		Nitrification	155.4800	4.89988	4
		Total	155.4800	4.43211	12
Total	No stress	Fish	160.4275	3.15073	4
		Algae	160.4275	3.15073	4
		Nitrification	160.4275	3.15073	4
		Total	160.4275	2.84994	12
	Stress	Fish	157.9275	8.34236	4
		Algae	157.9275	8.34236	4
		Nitrification	157.9275	8.34236	4
		Total	157.9275	7.54595	12
	Total	Fish	159.1775	5.98887	8
		Algae	159.1775	5.98887	8
		Nitrification	159.1775	5.98887	8
		Total	159.1775	5.72257	24

treatment = RAS without algae, stress = No stress, tank = Fish

Descriptive Statistics[a]

	N	Minimum	Maximum	Mean	Std. Deviation
TAN.1.00	2	.30	.36	.3300	.04243
TAN.2.00	2	8.83	9.15	8.9900	.22627
TAN.3.00	2	2.87	4.93	3.9000	1.45664
TAN.4.00	2	.39	.60	.4950	.14849
TAN.5.00	2	.84	1.12	.9800	.19799
NO2.1.00	2	.65	.67	.6600	.01414

NO2.2.00	2	11.27	11.69	11.4800	.29698
NO2.3.00	2	.64	1.05	.8450	.28991
NO2.4.00	2	.58	.59	.5850	.00707
NO2.5.00	2	.37	.37	.3700	.00000
NO3.1.00	2	148.94	154.00	151.4700	3.57796
NO3.2.00	2	105.79	111.58	108.6850	4.09415
NO3.3.00	2	100.69	102.20	101.4450	1.06773
NO3.4.00	2	161.63	164.20	162.9150	1.81726
NO3.5.00	2	159.37	164.59	161.9800	3.69110
Valid N (listwise)	2				

a. treatment = RAS without algae, stress = No stress, tank = Fish

treatment = RAS without algae, stress = No stress, tank = Algae

Descriptive Statistics[a]

	N	Minimum	Maximum	Mean	Std. Deviation
TAN.1.00	2	.30	.38	.3400	.05657
TAN.2.00	2	8.77	9.09	8.9300	.22627
TAN.3.00	2	3.04	4.86	3.9500	1.28693
TAN.4.00	2	.52	.64	.5800	.08485
TAN.5.00	2	.84	1.12	.9800	.19799
NO2.1.00	2	.55	.61	.5800	.04243
NO2.2.00	2	.39	.39	.3900	.00000
NO2.3.00	2	.67	1.03	.8500	.25456
NO2.4.00	2	.50	.56	.5300	.04243
NO2.5.00	2	.37	.37	.3700	.00000
NO3.1.00	2	148.52	155.01	151.7650	4.58912
NO3.2.00	2	105.52	109.79	107.6550	3.01935
NO3.3.00	2	100.49	106.59	103.5400	4.31335
NO3.4.00	2	161.71	163.47	162.5900	1.24451
NO3.5.00	2	159.37	164.59	161.9800	3.69110
Valid N (listwise)	2				

a. treatment = RAS without algae, stress = No stress, tank = Algae

treatment = RAS without algae, stress = No stress, tank = Nitrification

Descriptive Statistics[a]

	N	Minimum	Maximum	Mean	Std. Deviation
TAN.1.00	2	.36	.37	.3650	.00707
TAN.2.00	2	8.61	8.98	8.7950	.26163
TAN.3.00	2	2.80	4.88	3.8400	1.47078
TAN.4.00	2	.34	.42	.3800	.05657
TAN.5.00	2	.84	1.12	.9800	.19799
NO2.1.00	2	.62	.64	.6300	.01414
NO2.2.00	2	.39	.40	.3950	.00707
NO2.3.00	2	.63	1.03	.8300	.28284
NO2.4.00	2	.55	.55	.5500	.00000
NO2.5.00	2	.37	.37	.3700	.00000
NO3.1.00	2	151.88	155.06	153.4700	2.24860
NO3.2.00	2	104.53	112.68	108.6050	5.76292
NO3.3.00	2	102.44	102.55	102.4950	.07778
NO3.4.00	2	132.86	167.14	150.0000	24.23962
NO3.5.00	2	159.37	164.59	161.9800	3.69110
Valid N (listwise)	2				

a. treatment = RAS without algae, stress = No stress, tank = Nitrification

treatment = RAS without algae, stress = Stress, tank = Fish

Descriptive Statistics[a]

	N	Minimum	Maximum	Mean	Std. Deviation
TAN.1.00	2	.41	.53	.4700	.08485
TAN.2.00	2	12.50	13.56	13.0300	.74953
TAN.3.00	2	1.98	2.34	2.1600	.25456
TAN.4.00	2	.25	.42	.3350	.12021

TAN.5.00	2	.10	.15	.1250	.03536
NO2.1.00	2	.72	.99	.8550	.19092
NO2.2.00	2	1.42	16.70	9.0600	10.80459
NO2.3.00	2	7.26	7.93	7.5950	.47376
NO2.4.00	2	3.31	9.62	6.4650	4.46184
NO2.5.00	2	.45	.45	.4500	.00000
NO3.1.00	2	162.79	165.57	164.1800	1.96576
NO3.2.00	2	80.24	80.86	80.5500	.43841
NO3.3.00	2	75.47	79.50	77.4850	2.84964
NO3.4.00	2	119.90	140.89	130.3950	14.84217
NO3.5.00	2	158.63	168.91	163.7700	7.26906
Valid N (listwise)	2				

a. treatment = RAS without algae, stress = Stress, tank = Fish

treatment = RAS without algae, stress = Stress, tank = Algae

Descriptive Statistics[a]

	N	Minimum	Maximum	Mean	Std. Deviation
TAN.1.00	2	.16	.42	.2900	.18385
TAN.2.00	2	12.45	12.99	12.7200	.38184
TAN.3.00	2	2.02	2.26	2.1400	.16971
TAN.4.00	2	.31	.47	.3900	.11314
TAN.5.00	2	.10	.15	.1250	.03536
NO2.1.00	2	.67	.97	.8200	.21213
NO2.2.00	2	1.38	1.40	1.3900	.01414
NO2.3.00	2	7.19	7.98	7.5850	.55861
NO2.4.00	2	3.11	8.02	5.5650	3.47189
NO2.5.00	2	.45	.45	.4500	.00000
NO3.1.00	2	161.28	162.90	162.0900	1.14551
NO3.2.00	2	77.23	79.04	78.1350	1.27986
NO3.3.00	2	75.34	78.38	76.8600	2.14960
NO3.4.00	2	99.42	144.72	122.0700	32.03194
NO3.5.00	2	158.63	168.91	163.7700	7.26906
Valid N (listwise)	2				

a. treatment = RAS without algae, stress = Stress, tank = Algae

treatment = RAS without algae, stress = Stress, tank = Nitrification

Descriptive Statistics[a]

	N	Minimum	Maximum	Mean	Std. Deviation
TAN.1.00	2	.35	.48	.4150	.09192
TAN.2.00	2	11.57	13.46	12.5150	1.33643
TAN.3.00	2	1.95	2.28	2.1150	.23335
TAN.4.00	2	.19	.20	.1950	.00707
TAN.5.00	2	.10	.15	.1250	.03536
NO2.1.00	2	.71	.94	.8250	.16263
NO2.2.00	2	1.40	1.42	1.4100	.01414
NO2.3.00	2	7.18	7.96	7.5700	.55154
NO2.4.00	2	3.20	8.02	5.6100	3.40825
NO2.5.00	2	.45	.45	.4500	.00000
NO3.1.00	2	163.89	168.66	166.2750	3.37290
NO3.2.00	2	80.13	80.54	80.3350	.28991
NO3.3.00	2	75.41	78.38	76.8950	2.10011
NO3.4.00	2	99.42	144.72	122.0700	32.03194
NO3.5.00	2	158.63	168.91	163.7700	7.26906
Valid N (listwise)	2				

a. treatment = RAS without algae, stress = Stress, tank = Nitrification

treatment = RAS with algae, stress = No stress, tank = Fish

Descriptive Statistics[a]

	N	Minimum	Maximum	Mean	Std. Deviation
TAN.1.00	2	.14	.23	.1850	.06364
TAN.2.00	2	2.32	3.07	2.6950	.53033
TAN.3.00	2	.60	.60	.6000	.00000

TAN.4.00	2	.22	.34	.2800	.08485
TAN.5.00	2	.02	.16	.0900	.09899
NO2.1.00	2	.44	.49	.4650	.03536
NO2.2.00	2	.37	.38	.3750	.00707
NO2.3.00	2	.51	.69	.6000	.12728
NO2.4.00	2	.44	.48	.4600	.02828
NO2.5.00	2	.38	.41	.3950	.02121
NO3.1.00	2	129.36	138.97	134.1650	6.79530
NO3.2.00	2	105.15	114.40	109.7750	6.54074
NO3.3.00	2	97.70	103.67	100.6850	4.22143
NO3.4.00	2	149.06	151.78	150.4200	1.92333
NO3.5.00	2	157.07	160.68	158.8750	2.55266
Valid N (listwise)	2				

a. treatment = RAS with algae, stress = No stress, tank = Fish

treatment = RAS with algae, stress = No stress, tank = Algae

Descriptive Statistics[a]

	N	Minimum	Maximum	Mean	Std. Deviation
TAN.1.00	2	.35	.35	.3500	.00000
TAN.2.00	2	1.98	3.16	2.5700	.83439
TAN.3.00	2	.46	.46	.4600	.00000
TAN.4.00	2	.27	.43	.3500	.11314
TAN.5.00	2	.02	.16	.0900	.09899
NO2.1.00	2	.37	.38	.3750	.00707
NO2.2.00	2	.35	.37	.3600	.01414
NO2.3.00	2	.50	.70	.6000	.14142
NO2.4.00	2	.44	.44	.4400	.00000
NO2.5.00	2	.38	.41	.3950	.02121
NO3.1.00	2	129.85	141.23	135.5400	8.04688
NO3.2.00	2	104.65	110.03	107.3400	3.80423
NO3.3.00	2	97.97	103.06	100.5150	3.59917
NO3.4.00	2	122.16	170.51	146.3350	34.18861
NO3.5.00	2	157.07	160.68	158.8750	2.55266
Valid N (listwise)	2				

a. treatment = RAS with algae, stress = No stress, tank = Algae

treatment = RAS with algae, stress = No stress, tank = Nitrification

Descriptive Statistics[a]

	N	Minimum	Maximum	Mean	Std. Deviation
TAN.1.00	2	.34	.79	.5650	.31820
TAN.2.00	2	2.00	3.15	2.5750	.81317
TAN.3.00	2	.50	.50	.5000	.00000
TAN.4.00	2	.29	.61	.4500	.22627
TAN.5.00	2	.02	.16	.0900	.09899
NO2.1.00	2	.40	.48	.4400	.05657
NO2.2.00	2	.36	.37	.3650	.00707
NO2.3.00	2	.49	.68	.5850	.13435
NO2.4.00	2	.01	.44	.2250	.30406
NO2.5.00	2	.38	.41	.3950	.02121
NO3.1.00	2	130.34	139.59	134.9650	6.54074
NO3.2.00	2	104.25	113.60	108.9250	6.61145
NO3.3.00	2	98.98	103.51	101.2450	3.20319
NO3.4.00	2	145.68	161.67	153.6750	11.30664
NO3.5.00	2	157.07	160.68	158.8750	2.55266
Valid N (listwise)	2				

a. treatment = RAS with algae, stress = No stress, tank = Nitrification

treatment = RAS with algae, stress = Stress, tank = Fish

Descriptive Statistics[a]

	N	Minimum	Maximum	Mean	Std. Deviation
TAN.1.00	2	.16	.34	.2500	.12728
TAN.2.00	2	9.53	13.75	11.6400	2.98399

TAN.3.00	2	.69	1.77	1.2300	.76368
TAN.4.00	2	.20	.27	.2350	.04950
TAN.5.00	2	.25	.32	.2850	.04950
NO2.1.00	2	.58	.63	.6050	.03536
NO2.2.00	2	1.07	1.15	1.1100	.05657
NO2.3.00	2	1.07	4.28	2.6750	2.26981
NO2.4.00	2	.57	.59	.5800	.01414
NO2.5.00	2	.39	.42	.4050	.02121
NO3.1.00	2	130.63	133.68	132.1550	2.15668
NO3.2.00	2	71.07	72.45	71.7600	.97581
NO3.3.00	2	76.66	89.47	83.0650	9.05804
NO3.4.00	2	127.85	133.55	130.7000	4.03051
NO3.5.00	2	148.97	155.20	152.0850	4.40528
Valid N (listwise)	2				

a. treatment = RAS with algae, stress = Stress, tank = Fish

treatment = RAS with algae, stress = Stress, tank = Algae

Descriptive Statistics[a]

	N	Minimum	Maximum	Mean	Std. Deviation
TAN.1.00	2	.08	.29	.1850	.14849
TAN.2.00	2	7.56	13.76	10.6600	4.38406
TAN.3.00	2	.75	1.60	1.1750	.60104
TAN.4.00	2	.29	.39	.3400	.07071
TAN.5.00	2	.25	.32	.2850	.04950
NO2.1.00	2	.51	.55	.5300	.02828
NO2.2.00	2	1.08	1.08	1.0800	.00000
NO2.3.00	2	1.07	4.41	2.7400	2.36174
NO2.4.00	2	.47	.59	.5300	.08485
NO2.5.00	2	.39	.42	.4050	.02121
NO3.1.00	2	133.13	133.30	133.2150	.12021
NO3.2.00	2	68.99	68.99	68.9900	.00000
NO3.3.00	2	77.57	95.06	86.3150	12.36730
NO3.4.00	2	98.65	128.09	113.3700	20.81722
NO3.5.00	2	148.97	155.20	152.0850	4.40528

Valid N (listwise)	2				

a. treatment = RAS with algae, stress = Stress, tank = Algae

treatment = RAS with algae, stress = Stress, tank = Nitrification

Descriptive Statistics[a]

	N	Minimum	Maximum	Mean	Std. Deviation
TAN.1.00	2	.08	.83	.4550	.53033
TAN.2.00	2	8.99	13.56	11.2750	3.23148
TAN.3.00	2	.73	1.77	1.2500	.73539
TAN.4.00	2	.23	.23	.2300	.00000
TAN.5.00	2	.25	.32	.2850	.04950
NO2.1.00	2	.54	.55	.5450	.00707
NO2.2.00	2	1.09	1.17	1.1300	.05657
NO2.3.00	2	1.03	4.30	2.6650	2.31224
NO2.4.00	2	.53	.58	.5550	.03536
NO2.5.00	2	.39	.42	.4050	.02121
NO3.1.00	2	128.50	135.16	131.8300	4.70933
NO3.2.00	2	67.96	71.36	69.6600	2.40416
NO3.3.00	2	78.82	96.37	87.5950	12.40972
NO3.4.00	2	125.38	142.55	133.9650	12.14102
NO3.5.00	2	148.97	155.20	152.0850	4.40528
Valid N (listwise)	2				

a. treatment = RAS with algae, stress = Stress, tank = Nitrification
