# Supplementary figures and images for: Resistance and resilience of small-scale recirculating aquaculture systems (RAS) with or without algae to pH perturbation
Source: PLoS One. 2018 Apr 16;13(4):e0195862. doi: 10.1371/journal.pone.0195862 (PMC5901992; doi:10.1371/journal.pone.0195862)

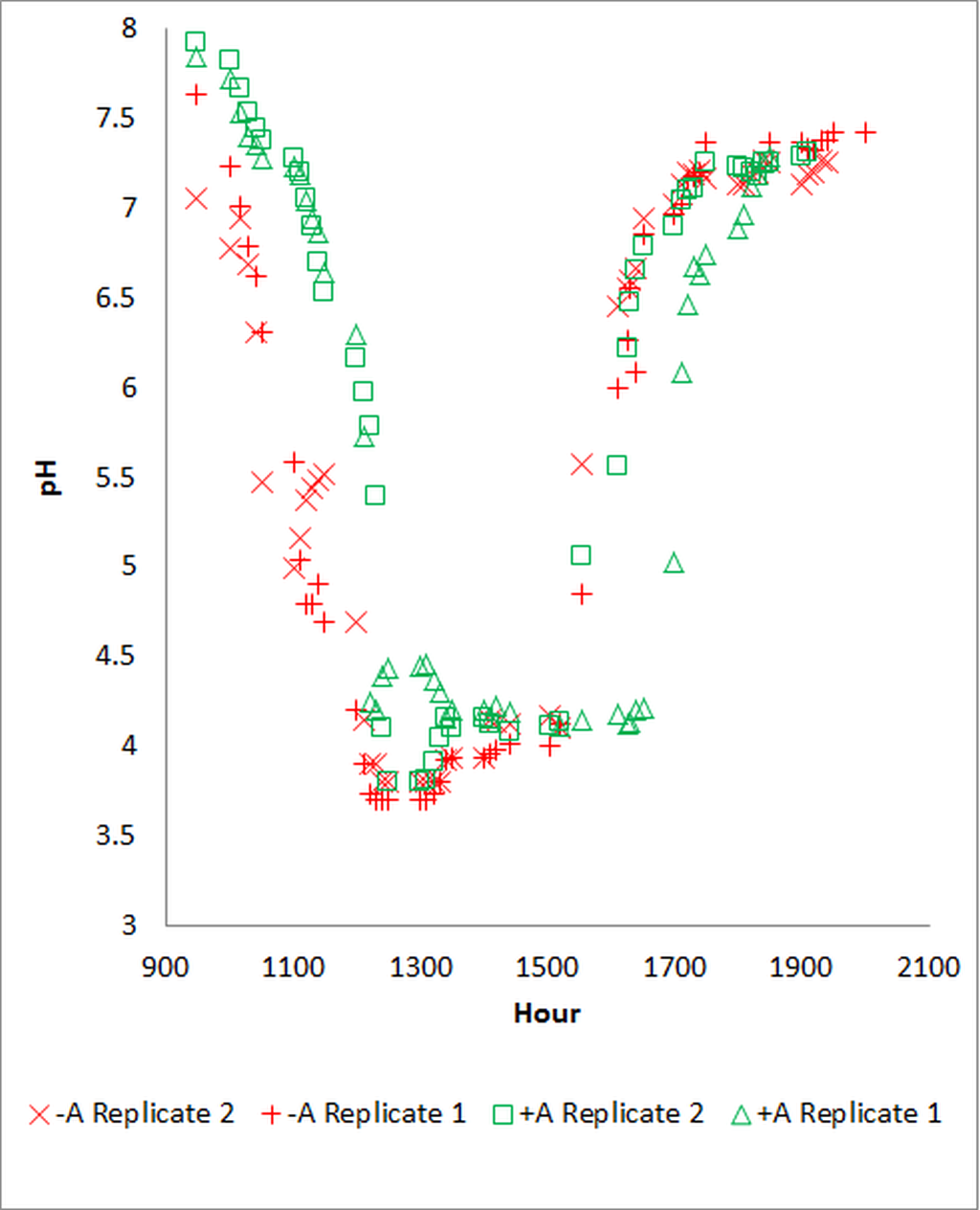

Supplement: S1 Fig — (TIF) [file pone.0195862.s005.tif]

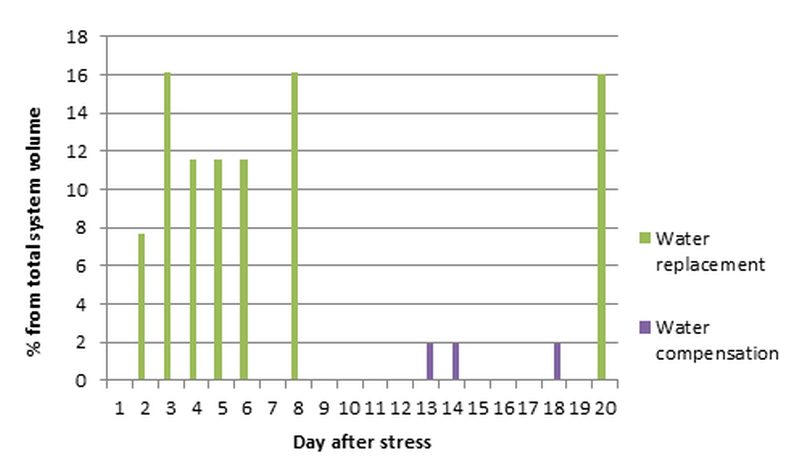

Supplement: S2 Fig — (TIF) [file pone.0195862.s006.tif]

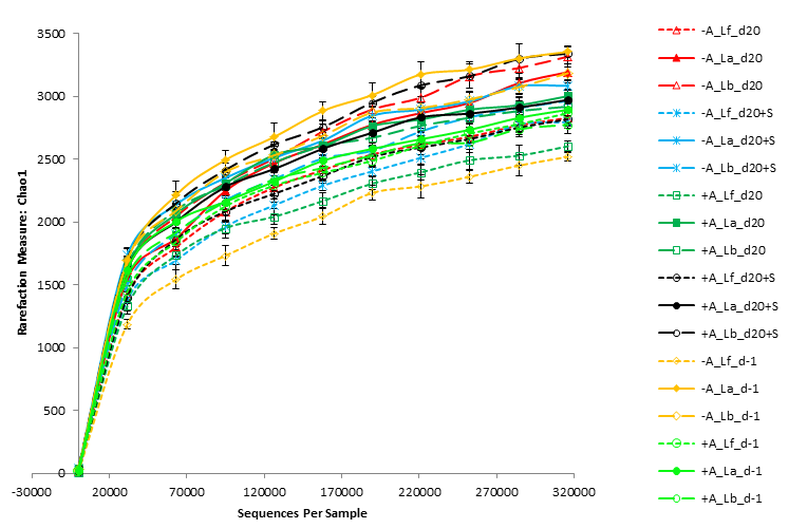

Supplement: S3 Fig — Samples are labeled with factors “algae”- with algae (+A), without algae (-A); “location”- fish (Lf), algae (La) and bio-filter (Lb) tanks; “day”- a day before stress (d-1), 20 days after stress (d20) and “stressor”- stressed (+S) and not stressed (-S). (TIF) [file pone.0195862.s007.tif]

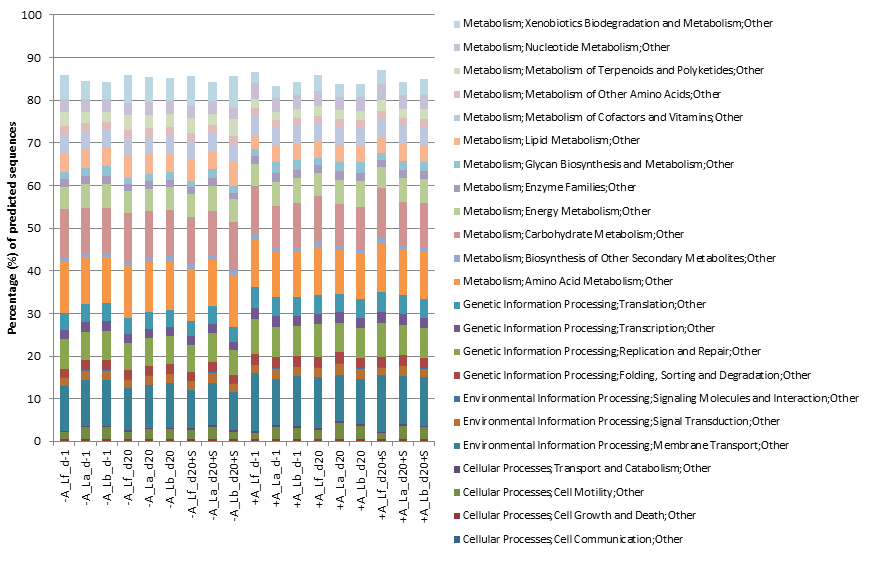

Supplement: S4 Fig — Samples are labeled with factors “algae”- with algae (+A), without algae (-A); “location”- fish (Lf), algae (La) and bio-filter (Lb) tanks; and stressor- stressed (+S) and not stressed (-S). (TIF) [file pone.0195862.s008.tif]

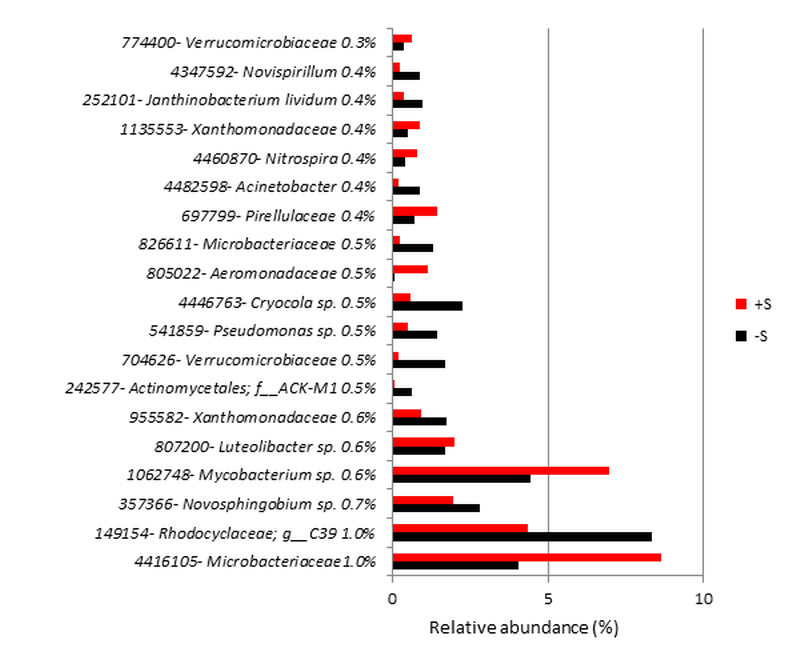

Supplement: S5 Fig — The graph shows the top 10% OTU which contributed to the total dissimilarity as given by SIMPER analysis. A number of percentage (%) written next to the identity of OTU denoted the % of contribution to the dissimilarity between +S and -S. (TIF) [file pone.0195862.s009.tif]

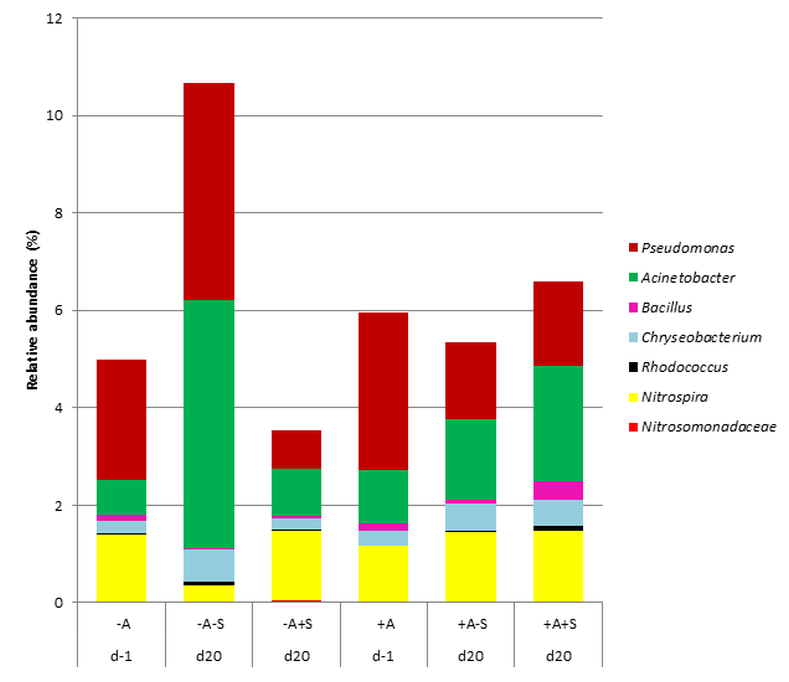

Supplement: S6 Fig — Bacteria which were able to perform autotrophic nitrification (Nitrosomonadaceae, Nitrospira) or heterotrophic nitrification and denitrification (Rhodococcus, Chryseobacterium, Bacillus, Acinetobacter, and Pseudomonas) identified in the recirculating aquaculture systems with (+A) and without algae (-A) a day before stress (d-1) and 20 days after stress (d20) which were stressed (+S) and not stressed (-S). (TIF) [file pone.0195862.s010.tif]
